# The Raman Map of the Human Cell

**DOI:** 10.1021/acs.analchem.5c02035

**Published:** 2025-07-28

**Authors:** A. M. Nowakowska, A. Pieczara, W. Korona, A. Borek-Dorosz, A. Adamczyk, P. Dawiec, P. Leszczenko, J. Orleanska, E. Machalska, B. Orzechowska, S. Orzechowska, K. Brzozowski, W. Krynicka, K. Majzner, K. Malek, M. Baranska

**Affiliations:** 1 Faculty of Chemistry, 37799 Jagiellonian University in Krakow, Gronostajowa 2, Krakow 30-387, Poland; 2 Jagiellonian Centre for Experimental Therapeutics (JCET), Jagiellonian University in Krakow, Bobrzynskiego 14, Krakow 30-348, Poland; 3 Doctoral School of Exact and Natural Sciences, Jagiellonian University in Krakow, Lojasiewicza 11, Krakow 30-348, Poland; 4 Institute of Nuclear Physics, Polish Academy of Sciences, Radzikowskiego 152, Krakow 31-342, Poland

## Abstract

Cell activity is governed by its subcellular compartments, each playing a specialized role essential to the overall function. Among various methods used for cell analysis, Raman microscopy is unique, enabling the study of single living cells at a submicron resolution. It is used for phenotyping of cells, monitoring the mechanisms of various stimuli or drugs, or tracking cell metabolism in real time. However, localizing specific organelles and identifying their components remain a challenge. Therefore, the “*Raman Map of the Cell*” aims to provide a reliable method for subcellular analysis and cataloging the Raman spectra of prominent cell organelles. In this study, we focused on endothelial cells due to their prevalence and widespread application in biomedical research. Using Raman imaging and advanced chemometric techniques, we mapped the subcellular landscape of a single endothelial cell. Individual organelles, including the nucleus, nucleoli, perinuclear area, mitochondria, lipid droplets, and cytoplasm, were localized, and their spectra were compared with those of reference compounds. As a result, we have established a comprehensive library of Raman profiles for cellular organelles, highlighting their marker bands for precise identification. The “*Raman Map of the Cell*” is discussed alongside relevant data from our previously published reviews on important molecules that occur naturally in biological systems, including proteins, lipids, and carbohydrates.

## Graphical abstract

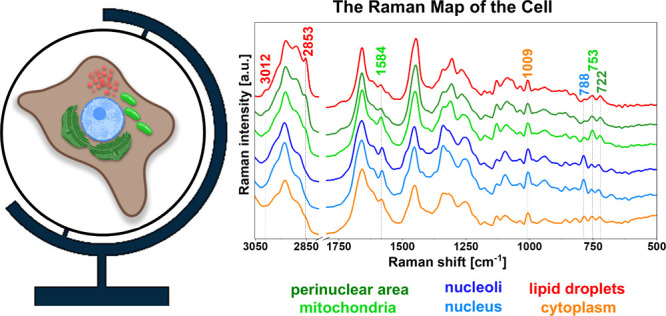

## Introduction

The human body comprises around 100 billion cells,^1^ divided into 400 types, each playing a specific role in human homeostasis. Subcellular compartments govern cell function and health, making cellular modeling vital for understanding disease progression.^2^ Single-cell studies of drug accumulation, cell phenotyping, and mapping of cellular metabolism form the foundation of individualized medicine. Studies focused on single organelles and their composition, structure, and function were also conducted in this article.

### Nucleus and Nucleoli

The nucleus is the center of genetic activity and plays a crucial role in cell function.^3^ The outer nuclear membrane blends with the rough endoplasmic reticulum (RER), while the inner nuclear membrane forms the organelle boundary. The nucleus contains chromatin (containing DNA and proteins), nucleoplasm (the internal fluid), multiple nucleoli, and mRNA associated with specific proteins.^4^ DNA in the nucleus exists in two forms, A and B, with B-DNA being the most common, occurring as a right-handed helix under physiological conditions.^5^

### Perinuclear Area

The endoplasmic reticulum (ER), the main component of the perinuclear area, is an indispensable organelle of all eukaryotic cells.^3^ It is traditionally categorized into RER and smooth ER (SER).^3^ One of the ER roles is the production of lipids required by various organelles.^3,6^ Furthermore, ER serves as a crucial hub for the trafficking, folding, translocation, and post-translational modifications of proteins, as well as for calcium signaling.^3,7,8^

### Mitochondria

This organelle is the “cell powerhouse”, producing energy through mitochondrial oxidative phosphorylation.^9^ Mitochondria also mediate interactions with other organelles and play a crucial role in numerous signaling pathways,^9^ including cell death regulation.^10^ Two independent outer and inner membranes separate the mitochondrion from the cellular cytoplasm. Energy production occurs through the electron transport chain (ETC), involving cytochrome c (cyt. c), ultimately generating adenosine triphosphate (ATP).^9^

### Lipid Droplets

Lipid droplets (LDs) are dynamic and versatile organelles that serve as lipid-rich sources in the cytoplasm of most eukaryotic cells.^11^ They actively participate in cellular processes such as lipid metabolism, energy homeostasis, and membrane trafficking.^12^ The versatility of LDs extends to their involvement in various diseases, including metabolic disorders, cancer, and infections.^12^ LDs consist of a core of neutral lipids, such as triacylglycerols (TAGs) and cholesterol esters (CEs), surrounded by a phospholipid monolayer with associated proteins.^11^

### Cytoplasm

The cell̀ interior is an intricate milieu, composed of a complex mixture of proteins, amino acids, fatty acids (FAs), carbohydrates, mRNA, inorganic salts, and water.^3^ This heterogeneous environment between the cell membrane and the nucleus is dynamic, with countless biochemical reactions and molecular interactions taking place.^13^ The cytoplasm is crucial for transporting and storing essential reaction substrates, maintaining cell shape, and regulating osmotic pressure.^14^ Ribosomes, the Golgi apparatus, and the ER are integral to the cytoplasm.^3^

Single-cell analysis methods, including flow cytometry, immunostaining, and sequencing, provide valuable information about individual cells’ overall behavior and function, including tracking carcinogenesis,^15^ inflammation,^16^ and cell–drug interactions.^17^ Nevertheless, these methods require labeling for the precise localization and quantification of specific targets at the subcellular level, particularly as the cellular interior is highly heterogeneous, regarding chemical diversity. Raman spectroscopy (RS) and its microscopic modalities address this limitation by combining chemical sensitivity with submicron resolution.

RS relies on the inelastic scattering of light as it interacts with sample molecules, providing detailed information about their chemical structure. Raman confocal microscopy enables label-free tracking of cellular processes in single living cells with high spatial resolution^18^ and in real time.^19^ Because of the large number of cell constituents, a cell’s Raman spectra are complex, but chemometric analysis enables the detection of organelles and the determination of their composition. This approach, although common, depends on reliable band assignments to clearly interpret biological mechanisms.

Numerous studies have focused on subcellular analysis using Raman microscopy, aiming to identify marker bands or spectral profiles characteristic of specific organelles, utilizing, for example, single band analysis,^20^ chemometric methods,^20^ immunofluorescence imaging,^21^ or transcriptomics analysis.^22^ Additionally, attempts have been made in the literature to construct databases of Raman spectra of cellular components and to systematize band assignments by comparing them with reference spectra.^23,24^ These studies greatly contributed to understanding the complexity of the Raman spectra of single cells. However, many were limited by the use of fixed cells, which hindered mitochondrial identification,^23^ by a lack of a comparison with a database of spectra from various endogenous cellular components,^20,25^ or by comparing reference spectra with averaged whole-cell spectra, reducing subcellular resolution.^24^ Therefore, we aimed to take a step further by combining Raman imaging of a single live cell, enabling the localization of individual organelles, with a comparison of cellular spectra to an extensive database of reference compound spectra to assign Raman bands specific to organelles and generate a so-called “*Raman Map of the Cell*”.

This paper is an original work on subcellular high-resolution analysis of single cells, including a comprehensive discussion of the previously published work on Raman microscopy-based cell analysis. The paper refers to our previously published reviews on biologically important chemical components, including proteins,^26^ lipids,^27^ and carbohydrates.^28^ As a result, we present a detailed analysis of a single cell together with a library of Raman spectra of cell components and organelles with band assignments. The analysis was performed on endothelial cells (ECs), which participate in numerous critical biological processes and interactions related to maintaining homeostasis in the organism.^29^ These cells were used as a model to distinguish characteristic cellular structures, including the nucleus, nucleoli, perinuclear area (with contributions from ER), mitochondria, LDs, and cytoplasm.

## Materials and Methods

### Raman Imaging

The human aortic EC cell line (HAEC, Lonza) is the *in vitro* model selected to examine the cellular composition. Raman imaging of living HAEC cells was performed using a confocal Raman microscope, WITec Alpha 300 (WITec GmbH), operating at 532 nm with a laser power of approximately 30 mW (measured before the objective). Approximately 40 cells were measured (from three different measurement sets). Cells were imaged using a 60× water immersion objective (Nikon Fluor, NA = 1) with a step size of 0.5 μm and an integration time of 0.1 s per spectrum. Data processing was performed using WITec Project Plus software as described in the SI. In the article, we present K-means cluster (KMC) results for selected cells. The analyzed mean spectra of the individual subcellular classes originate from a single, representative cell where distinct organelles were clearly observable based on their characteristic Raman fingerprints.

### Raman Measurements of Reference Compounds

The reference compounds were measured in the solid state (except amino acids measured in solution) and are listed in Table S1. The samples were placed on a CaF_2_ window and illuminated through an air Olympus MPLAN objective (100×/0.9 NA) or a Zeiss W N-achroplan objective (20×/0.5 NA). At least three Raman spectra were recorded from randomly chosen spots with a 0.5 s integration time, 10 accumulations, and a laser power of 50 mW. More details are provided in the SI.

## Results and Discussion

In the first part of the cell analysis, k-means cluster analysis (KMC) was performed on Raman images of selected representative ECs, enabling the identification of individual cell organelles and providing information about their spatial distribution and average spectra. The localization of organelles, key to cell function, which were presented in the KMC map, was compared with fluorescence imaging. In the second part, we compared the mean spectra of cellular organelles from a selected representative cell (Figure S1) with those of reference compounds, allowing us to determine unique spectral signatures that can serve as molecular fingerprints for each subcellular component.

KMC analysis identified key cellular structures: the nucleus, nucleoli, perinuclear area (with a contribution of ER), mitochondria, LDs, cytoplasm, and cell membrane (Figure 1 and Figure S1). Each spectrum (Figure 2A and Figure S2) was assigned to a particular subcellular compartment based on the presence of a set of characteristic Raman bands (annotated with corresponding colors, Table 1) that serve as molecular fingerprints for endogenous biomolecules within each organelle. The color-coded KMC maps in Figure 1 and Figure S1 convey the overall cell morphology and delineate the subcellular distribution of individual organelles. KMC analysis was confirmed by Raman maps generated by integrating organelle-specific bands as well as by intensity KMC, and fluorescence images highlighting crucial organelles using targeted labeling (nuclei were labeled using Hoechst 33342, LDs were labeled using HSC LipidTOX, and mitochondria were labeled using MitoTracker). However, due to the colocalization of various biomolecules within cellular structures, single-band hyperspectral analysis is usually insufficient for comprehensive examination. Therefore, to identify selected structures in cells, the presence of a set of characterization bands was always verified, rather than the intensities of a single band or the entire spectrum.

**Figure 1:**
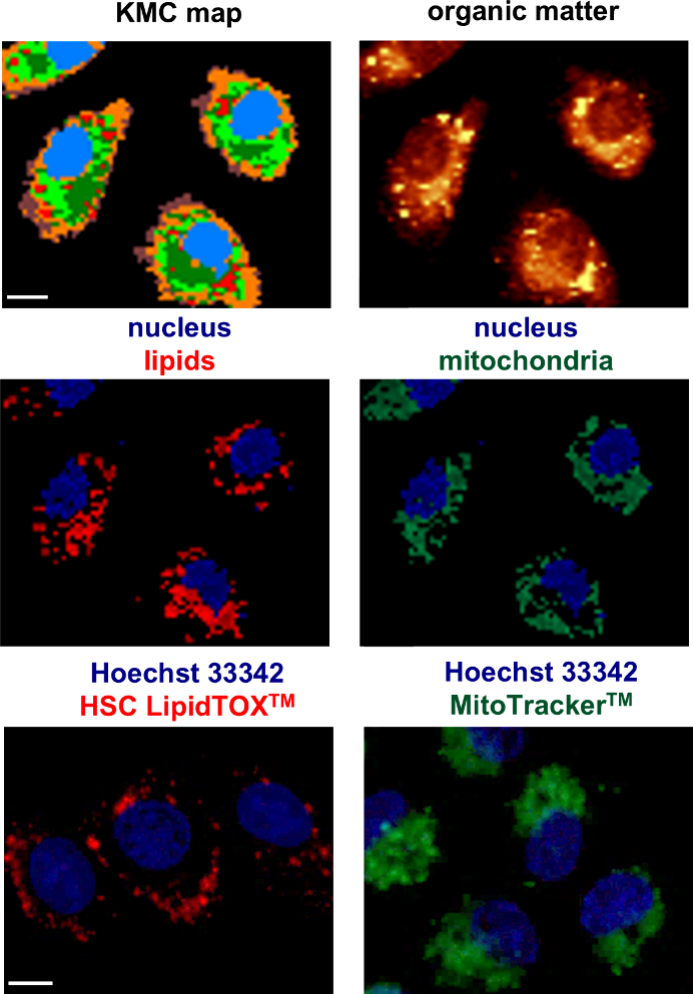
KMC analysis of HAEC cells showing the distribution of different subcellular compartments, including the nucleus (azure), perinuclear area (green), mitochondria (vivid green), LDs (red), cytoplasm (orange), and cell membrane (dark brown) with representative Raman image integrated over 2835–2935 cm^−1^ (organic matter), visualizing the morphology of cells (top row). Intensity images of KMC analysis showing the localization of nuclei (blue) and LDs (red), as well as the nuclei (blue) and mitochondria (green) (middle row), were compared with counterstained fluorescence images showing the distribution of the same organelles (bottom row; nuclei were labeled using Hoechst 33342, LDs were labeled using HSC LipidTOX, and mitochondria were labeled using MitoTracker). Scale bar: 10 μm.

**Figure 2:**
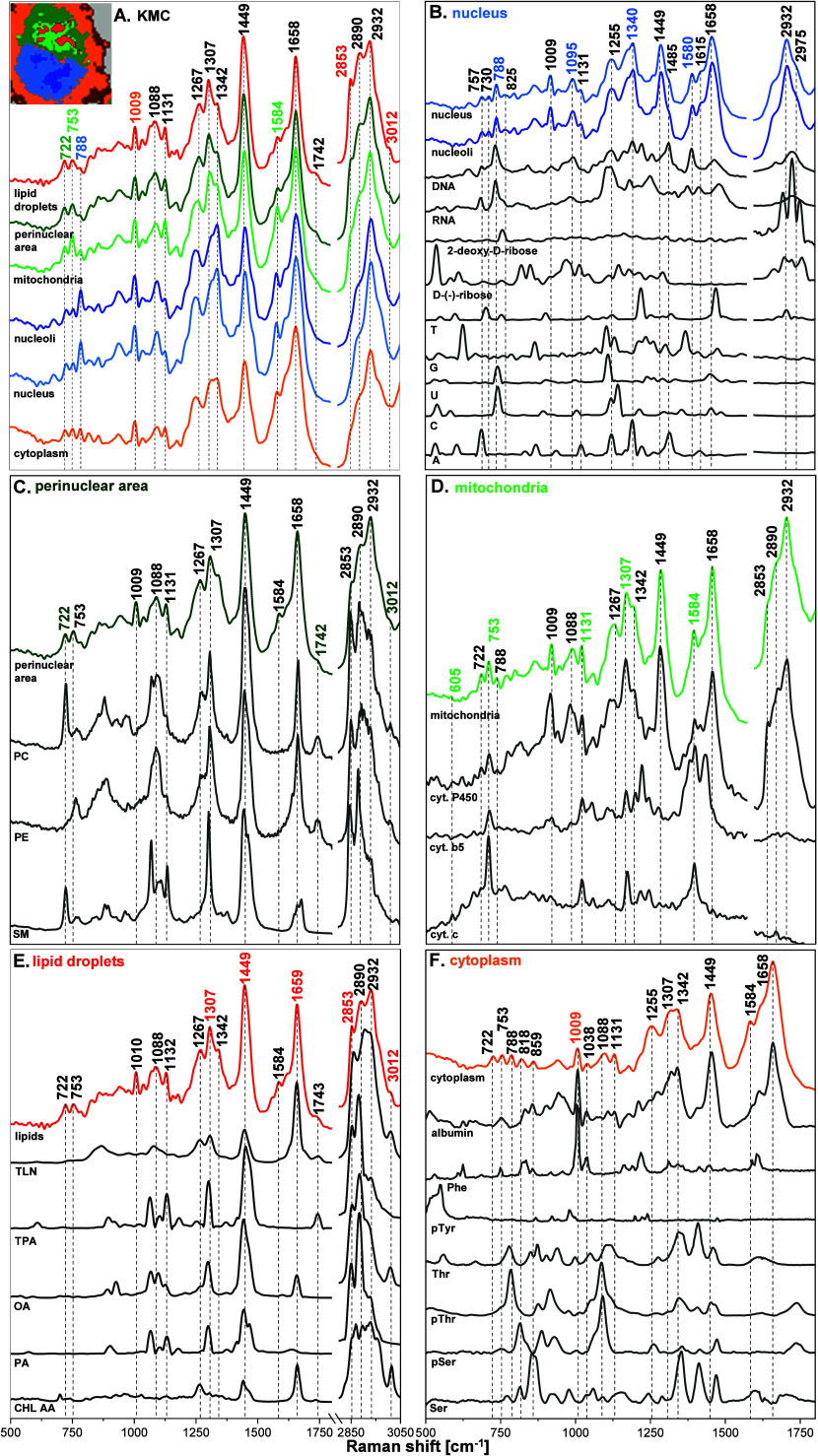
(A) The average Raman spectra of subcellular components - nucleus (azure), nucleoli (blue), perinuclear area (green), mitochondria (vivid green), LDs (red), and cytoplasm (orange). (B) The spectra of the nucleus and nucleoli in comparison with the spectra of reference compounds: calf thymus DNA, Torula yeast RNA, deoxy-2-d-ribose, d-(−)-ribose, and nucleobases (A, C, G, T, U). (C) The spectrum of the perinuclear area in comparison with reference standards: phosphatidylcholine (PC), phosphatidylethanolamine (PE), and sphingomyelin (SM). (D) The spectrum of mitochondria in comparison with selected standards: cytochrome P450 (cyt. P450), cytochrome b5 (cyt. b5), and cytochrome c (cyt. c). (E) The spectrum of the LDs in comparison with selected standards: trilinolein (TLN), tripalmitin (TPA), oleic acid (OA), palmitic acid (PA), and cholesteryl arachidonate (CHL AA). (F) The spectra of the cytoplasm (orange) and cell membrane in comparison with the spectra of O-phospho-l-tyrosine (pTyr), O-phospho-l-threonine (pThr), l-threonine (Thr), O-phospho-l-serine (pSer), and l-serine (Ser) in aqueous solution as well as with the spectra of albumin and l-phenylalanine (Phe) in powder. The spectra in the fingerprint region (1800–500 cm^–1^) are magnified three times for clarity, except for nucleobases.

**Table 1:**
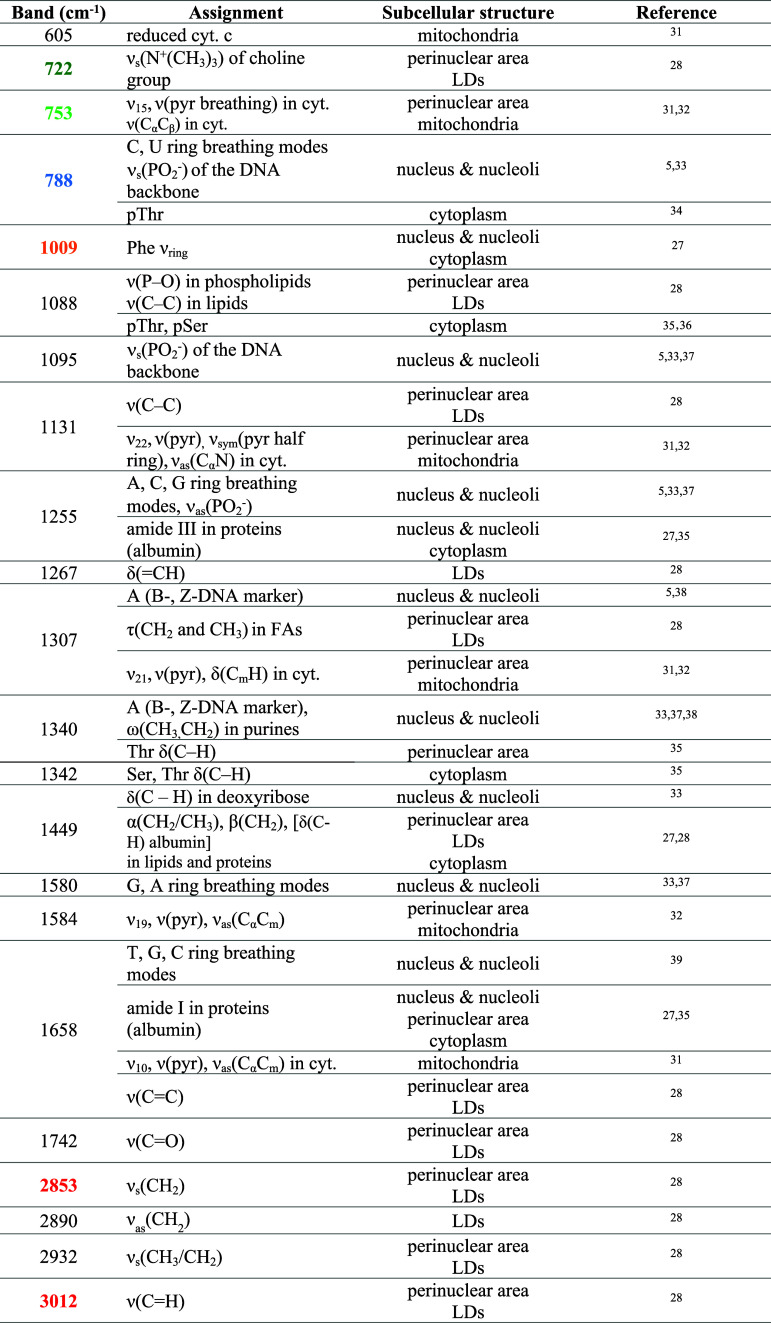
Characteristic Raman Bands of Subcellular Structures with the Corresponding Assignments^a^

a Abbreviations: α – scissoring; δ – deformation; ω – wagging; τ – twisting; ν – stretching (s – symmetric; as – asymmetric), A – adenine, C – cytosine, G – guanine, T – thymine, U – uracil, pyr – porphyrin ring, cyt. – cytochromes, p – phosphorylated, Ser – l-serine, Thr – l-threonine, Phe – l-phenylalanine, LDs – lipid droplets, FAs – fatty acids. Bold band positions indicate key structural markers.

The nucleus, located centrally in the cell, contains nucleoli (blue classes in the KMC map, Figures 1, 2A and Figures S1 and S2). Despite the similar composition, nucleoli were distinguished from the nucleus based on the higher intensity of Raman bands associated with nitrogenous bases (notably at 788 cm^–1^) and the PO_2_^–^ group (bands at 1095 and 1255 cm^–1^). Surrounding the nucleus is the perinuclear area, segregated by KMC into two distinct subregions (green classes in the KMC map, Figures 1, 2A, Figures S1 and S2). The high cytochrome content differentiates the outer perinuclear region, indicating the presence of mitochondria (vivid green) as shown by fluorescence imaging. Mitochondria exhibit intense Raman features that can be attributed to heme proteins at 753, 1131, 1307, and 1584 cm^–1^. The central perinuclear region is a lipid-rich structure and contains LDs (red class), identified by prominent signatures of saturated (2853 cm^–1^) and unsaturated (3012 cm^–1^) fatty acids. As shown in Figure 1 and Figure S1, the perinuclear area and LDs occupy a significant part of the cytoplasm, which was confirmed by fluorescence microscopy. The remaining spectra of the Raman map of the ECs are attributed to the cytoplasm and cell membrane based on their elevated protein content. However, the spectrum of the membrane had a lower intensity than the spectrum of the cytoplasm (Figure S2). Detailed vibrational band assignments and comparisons with the reference spectra are discussed below and are presented in Table 1.

### Nucleus and Nucleoli

Figure 2B and Figures S2–S4 present the average Raman spectrum of the nuclear area in the HAEC cell, compared to the spectra of DNA from the calf thymus, RNA from Torula yeast, deoxy-2-d-ribose, d-(−)-ribose, and nucleobases.

In the Raman spectra of the nucleus and nucleoli, the 1658 cm^–1^ band reflects amide I vibrations of histones in chromatin but may also involve thymine (T), guanine (G), and cytosine (C).^38^ Raman bands at 1307, 1342, and 1580 cm^–1^ indicate the presence of purine bases, particularly adenine (A) and G, through their ring breathing modes.^5,32,35,36,37^ A Raman feature at approximately 1449 cm^–1^, assigned to δ­(C–H), can be attributed to the sugars in DNA, histones, and deoxyribose.^26^ Characteristic bands associated with the ν­(PO_2_^–^) of the DNA backbone were observed at 1095 and 1255 cm^–1^.^5,32,36^ However, it should be noted that the 1267 cm^–1^ band may also incorporate contributions from the breathing vibrations of A, C, and G rings^5,32,36^ and the amide III vibrations of the histone proteins.^26,34^ A phenylalanine (Phe) band at 1009 cm^–1^ adds to the nuclear protein signals within the nucleus.^26,34^ The most characteristic band of the nucleus is located at 788 cm^–1^ and is primarily assigned to the breathing vibration of the C ring and the vibration of the PO_2_^–^ group.^5,32^ The main difference between the spectra of the nucleus and the nucleoli is the increased intensity of bands at 788, 1095, 1255, 1340, and 1580 cm^–1^, associated with the nucleobases and the PO_2_^–^ contributions.

While both DNA and RNA show a band at 788 cm^–1^, the 1580 cm^–1^ band is unique to DNA, and RNA displays distinct bands at 810–820 cm^–1^ due to its structure and function.^39^ Monitoring RNA-specific bases, such as uracil (U), is challenging due to overlapping with DNA bands near 785 cm^–1^, and although DNA and RNA share phosphate bands (∼1090 cm^–1^), RNA shows shifts from hydrogen bonding and backbone conformation. These markers help track RNA structural changes under stress or regulation.

The cell nucleus has been extensively studied using RS, which has provided information on its composition, structure, and dynamics and thus a better understanding of cellular processes and the mechanism of disease development. Raman marker bands of the nucleus have been used to differentiate normal and cancerous cells, to analyze differences in nuclear structure and organization,^40^ including single chromosomes,^41^ to quantify DNA in cell nuclei,^42^ and to track transcriptomic activity.^43^ DNA conformations are highly sensitive to environmental conditions, and transitions among the A-, B-, and Z-forms can be readily monitored using RS, as described in the SI.

### Perinuclear Area

Figure 2C and Figures S2 and S5 illustrate a comparative analysis of the Raman spectrum of the perinuclear area of living ECs, encompassing both RER and SER, alongside the spectra of the reference components selected based on their known abundance in cell membranes,^44^ i.e., phosphatidylcholine (PC), phosphatidylethanolamine (PE), and sphingomyelin (SM).

The Raman spectrum of the perinuclear area, along with reference spectra of PC, PE, and SM, reveals lipid-specific bands at 2853 cm^–1^ (ν_s_(=CH_2_)) and 3012 cm^–1^ (ν­(=CH)).^27^ A low-intensity band at 1742 cm^–1^ (ν­(C=O))^27^ appears in PE and PC spectra, while PC and PE spectra exhibit a 1307 cm^–1^ band (τ­(CH_2_ and CH_3_)).^27^ Additional shared features include bands at 1449 cm^–1^ α­(CH_2_/CH_3_) and 1658 cm^–1^, attributed to ν­(C=C) and amide I modes, associated with both lipids and proteins.^26,27^ Perinuclear regions also show strong amide I (1650–1670 cm^–1^) and amide III (1240–1300 cm^–1^) bands, reflecting protein processing in the ER, along with a phenylalanine band at 1009 cm^–1^, indicative of high protein turnover in metabolically active zones.

While the nucleus holds the highest concentration of DNA, the perinuclear region can contain RNA. Raman signals around 780–800 cm^–1^ and 1090 cm^–1^ are typically attributed to ν_s_(PO_2_^–^) of nucleic acids. Notably, low-intensity bands of cyt. c are observed in the Raman spectrum of the perinuclear area (at 753, 1131, 1307, and 1584 cm^–1^; Figure 2C). The most characteristic Raman bands of ER found in the perinuclear area are those related to phospholipids,^27^ specifically the bands at 722 cm^–1^, which corresponds to the (ν_s_(N^+^(CH_3_)_3_) mode of the choline group, 1088 (ν­(P–O)), and 1131 cm^–1^ (ν­(C*–*C)). Perinuclear organization is linked to gene expression and transport, with chemical changes reflecting stress responses like unfolded proteins or lipid imbalances—detectable by RS.^44^ Shifts in lipid saturation (∼2855/∼3010 cm^–1^) reveal metabolic states, and decoding ER-specific spectra offers insights into lipid–protein dynamics, with most studies focusing on drug-induced ER stress.^45^

### Mitochondria

Figure 2D and Figures S2 and S6 present the KMC-derived Raman spectrum of the cytochrome-rich ER of HAEC cells, alongside Raman spectra of cyt. c, cyt. P450, and cyt. b5—the main hemeproteins found in mitochondria.^33^

With an excitation laser line near the Soret/Q-band, the spectral fingerprints of cytochromes can be probed using resonance RS (RRS),^46^ as described in the SI. Under 532 nm laser excitation, the four porphyrin-derived bands are used to analyze the cytochrome content in cells, i.e., 753, 1131, 1307, and 1584 cm^–1^ (Figure 2D).^30–32,36^ These bands are predominant in the reference spectra of cytochromes (cyt. c, P450, and b5), though their relative intensities vary by type and are not easily distinguishable in whole-cell spectra.^30^ The band at 753 cm^–1^ is widely used as a spectral marker of mitochondria, as it allows the observation of the cytochrome signal without interference from other biomolecule signals, such as lipids, proteins, or genetic material.^30,31^

RRS provides insight into the oxidation and spin states^47^ of the heme iron, shedding light on mitochondrial bioenergetics and function. Changes in the redox state of cyt. c lead to shifts in porphyrin ring vibration modes^48^ and alterations in the intensities of hemoprotein peaks.^30^ Analysis of the redox state of cytochrome using RS is crucial, as it can indicate changes related to mitochondrial dysfunction,^49^ activity,^50^ and apoptosis.^51^

Mitochondria contain their own circular DNA, though in a much lower quantity compared to nuclear DNA. Phosphate-backbone signatures (at 1088 and 788 cm^–1^) may appear in mitochondrial regions, but these signals are generally weaker than those from nuclear DNA.

### Lipid Droplets

Figure 2E andFigures S2 and S7 show the KMC-derived Raman spectrum of LDs in HAEC cells together with the Raman spectra of trilinolein (TLN), tripalmitin (TPA), oleic acid (OA), palmitic acid (PA), and cholesteryl arachidonate (CHL AA).

LDs are primarily composed of TAGs and CEs. Therefore, the Raman spectra of LDs show bands corresponding mainly to C*–*O, C*–*C, C=C, and C*–*H vibrations.^27^ LD spectra typically exhibit a distinctive spectral marker of FAs and LDs at 2853 cm^–1^ (ν_s_(CH_2_)),^27^ as well as bands at 2890 (ν_as_(CH_2_)) and 2932 (v_s_(CH_3_/CH_2_)). These bands appear with varying intensities in the FAs and TAG reference spectra. Other lipid-specific Raman bands at 1267 (δ­(=CH)), 1307 (τ­(CH_2_)), 1449 (α­(CH_2_), 1658 (ν­(C = C)), and 3012 cm^–1^ ν­(=CH), visible in the spectra of the unsaturated lipids TLA, OA, and CHL AA, enable semiquantitative assessment of unsaturation levels in cells by analysis of bands^27,52^ (1307/1267, 1658/1449). However, because the 1267 and 1307 cm^–1^ bands partially overlap with other Raman signals (especially from proteins), these ratios can be challenging to interpret.^53^ A key band at 1742 cm^–1^ (ν­(C=O)) reflects the ester bonds in TAGs and CEs, distinguishing them from free FAs and enabling the tracking of lipid esterification by RS.^54^

The bands at 722 and 1088 cm^–1^, corresponding to ν­(P*–*O) and symmetric stretching vibrations of the choline group N^+^(CH_3_)_3_, serve as markers of membrane lipids, as discussed in the section on perinuclear area analysis.^27^ Alongside lipids, protein features can also be recognized in the LD spectra, particularly the amide I, amide III, and Phe (1009 cm^–1^) signals.^26,34^

RS is widely used in lipid analysis due to its large scattering cross-section of its long hydrocarbon chains. Consequently, RS has provided valuable information on LD distribution, size, and composition and enabled the monitoring of lipid metabolic dynamics, such as changes in unsaturation, esterification, and cholesterol content.^54,55^ An increased number of LDs and their size have been recognized as a hallmark of oncogenesis, reflecting enhanced lipid uptake and lipid synthesis in cancer cells.^56^ The degree of lipid unsaturation has been linked with endothelial inflammation.^54^

### Cytoplasm

The Raman spectrum of the cytoplasm (Figure 2F and Figure S2) of the HAEC cell shows bands associated with albumin, O-phospho-l-tyrosine (pTyr), O-phospho-l-threonine (pThr), l-threonine (Thr), O-phospho-l-serine (pSer), l-serine (Ser), and phenylalanine (Phe). However, in the complex cytoplasmic environment, many amino acid-derived bands are faint or overlap with other biomolecular features, as described in detail in the SI (Figures S8 and S9, Table S2).

The spectrum of the cytoplasm exhibits characteristic bands associated with proteins, including amide III (1255 cm^–1^) and amide I (1658 cm^–1^), which closely match those observed in the spectrum of albumin. The positions and deconvolution of amid III and amide I bands provide information about proteins’ secondary structure (see the SI). However, most studies of protein secondary conformation are based on isolated proteins, as it is challenging to determine these structures in complex systems such as cells or tissues.^57^ Also, the fixation method can significantly alter the molecular architecture of biological samples, especially proteins (see the SI).

Among the amino acids, Phe (Figure 2F) is the most readily detectable by RS. It is unequivocally identified by its 1009 cm^–1^ band, corresponding to the ring-breathing vibration in the monosubstituted benzene ring.^26,34^ However, instead of the specific band at 1009 cm^–1^, Phe is widely analyzed using an additional band at 1038 cm^–1^, which is associated with in-plane ring bending (β­(C–H)).^26^ Another amino acid that exhibits characteristic bands in the cytoplasmic spectrum is Tyr. The most commonly used spectral markers of Tyr are 835 and 850 cm^–1^ bands, known as the Fermi doublet.^26,34^ Phosphorylation of the Tyr leads to the collapse of the intensity of the Fermi doublet, making it detectable at 825 cm^–1^.^26^

As the cytoplasm contains both proteins and lipids, careful spectral analysis of biological samples is necessary. The amide I band overlaps with bands originating from the C=C vibration of unsaturated lipids, while amide III, associated with N–H bending and C–N stretching, may overlap with the C=C vibrations at 1260 cm^–1^.^27^ In some cases, weak contributions from nucleic acid vibrations may also fall within or near the amide III bands, further complicating the spectral analysis. The band at 1449 cm^–1^ (δ­(C–H), α­(CH_2_/CH_3_), β­(CH_2_))^26^ serves as a valuable marker of proteins, but it overlaps with a prominent CH_2_ bending band from lipids near 1440 cm^–1^. In contrast, some components of the cytoplasm, such as cell membranes, are difficult to detect in a label-free manner (see the SI).

## Conclusions

We have demonstrated that RS imaging of cells, supported by reference spectra analysis, enables the reliable interpretation of cells’ complex chemical environments. By collecting and systematizing Raman markers for the identification of individual organelles, we created the “*Raman Map of the Cell*”. We have built a comprehensive library of organelles and their assigned Raman spectra. We have distinguished key organelles in the cell, including the nucleus, nucleoli, the ER-rich perinuclear area, mitochondria, LDs, and the cytoplasm, which were characterized in detail from a spectroscopic standpoint, and correlated with their specific biological functions.

The Raman markers for individual organelles are summarized as follows:

1. Nucleus: Nuclei are identified by Raman features of DNA bases, including a highly characteristic band at 788 cm^–1^, derived from pyrimidine bases and PO_2_^–^ groups. Additional nucleotide base bands appearing at 730, 1340, 1485, 1580, and 1658 cm^–1^ can also be observed in the spectrum of the nuclear area. Signatures of PO_2_^–^ groups of the DNA backbone are seen at 1095 and 1255 cm^–1^, whereas protein-specific bands include the Phe ring vibrations (1009 cm^–1^), amide III (1255 cm^–1^), and amide I (1658 cm^–1^).

2. Perinuclear area: The key spectral features of the perinuclear area originate from lipids at 1307 and 2853 cm^–1^, lipids and proteins at 1449, 1658, and 2932 cm^–1^, and phospholipids and choline-containing lipids at 722 and 722 cm^–1^. In addition, the signals from cytochromes at 753 and 1131 cm^–1^ can be seen.

3. Mitochondria: Mitochondria are relatively easy to identify, especially when taking advantage of RRS. The intense bands of cytochromes assigned to the pyrrole rings of the heme core are observed at 753, 1131, 1307, and 1584 cm^–1^. Cytochrome signals may overlap with other biomolecules, but their identification is reliable when four characteristic bands are present.

4. LDs: The main components of LDs are TAGs, including esterified FAs, with their Raman profiles marked by C*–*O, C*–*C, and C=C vibrations. The 1307 and 1449 cm^–1^ bands always accompany the high-wavenumber bands at 2853, 2890, and 2932 cm^–1^. The spectrum of LDs reveals both the presence of lipidic structures and their unsaturation (at 1267, 1658, and 3012 cm^–1^) or esterification (at 1742 cm^–1^). Phospholipids and choline-containing lipids feature marker bands at 722 and 1088 cm^–1^, respectively.

5. Cytoplasm: Due to the high protein content, the Raman spectra of the cytoplasm are predominantly characterized by the Phe band at 1009 cm^–1^, the amide III band at 1255, and the amide I band at 1658, reflecting the protein secondary structure.

## Supplementary Material



## Data Availability

Raw measurement data are available here: https://doi.org/10.57903/UJ/EDV49Z.

## Notes

Original spectra, Raman band assignments, and organelle-specific analyses (PDF)
