# Supplementary material for: The Raman Map of the Human Cell
Source: Anal Chem. 2025 Jul 28;97(30):16374–82. doi: 10.1021/acs.analchem.5c02035 (PMC12332844; doi:10.1021/acs.analchem.5c02035)
Supplement: Supplementary file 1 [file ac5c02035_si_001.pdf]

Supporting Information

The Raman Map of the Human Cell

A. M. Nowakowska[1], A. Pieczara[1,2], W. Korona[1,3], A. Borek-Dorosz[1], A. Adamczyk[1], P. Dawiec[1,3], P. Leszczenko[1,3], J. Orleanska[1,3,4], E. Machalska[2], B. Orzechowska[1], S. Orzechowska[1], K. Brzozowski[1], W. Krynicka[1], K. Majzner[1]*, K. Malek[1]*, M. Baranska[1]*

[1]*Jagiellonian University, Faculty of Chemistry, Gronostajowa 2, 30-387, Krakow, Poland*

[2] *Jagiellonian University, Jagiellonian Centre for Experimental Therapeutics (JCET), Bobrzynskiego 14, 30-348 Krakow, Poland*

[3] *Jagiellonian University in Kraków, Doctoral School of Exact and Natural Sciences, Lojasiewicza 11, 30-348, Krakow, Poland*

[4] *Institute of Nuclear Physics, Polish Academy of Sciences, Radzikowskiego 152, 31-342 Kraków, Poland*

Corresponding Authors: K. B. M. : katarzyna.b.majzner@uj.edu.pl; K. M.: kamilla.malek@uj.edu.pl; M. B.: m.baranska@uj.edu.pl

Table of contents

A list of abbreviations 2
Supplementary Materials and Methods 3
- Cells culture 3
- Data Analysis 3
- Raman measurements of references and data analysis 4
- The list of reference compounds 4
- Fluorescence imaging 5

The identification of individual cell organelles based on Raman imaging 5
The average spectra of subcellular components 6
Nucleus & nucleoli 6
- Spectroscopic analysis of DNA conformations 7

Perinuclear area 9
Mitochondria 11
- Resonance Raman Spectroscopy of hemoproteins 11
- Spectroscopic analysis of cytochromes 11

Lipid droplets 12
Cytoplasm 13
- Spectroscopic analysis of the secondary structure of proteins 14
- Spectroscopic analysis of the structure of albumin 14
- Raman bands of amino acids 14
- The influence of fixation on the molecular structure of proteins 14
- Detection of lipid membranes using Raman spectroscopic methods 15

A list of figures and tables

Figure S1. *KMC results of HAEC cell with representative Raman images.*

Figure S2. *The average spectra of subcellular components.*

Figure S3. *Average Raman spectra of the nucleoli and nucleus of HAEC cells in comparison with spectra of reference compounds (DNA and RNA).*

Figure S4. *Average Raman spectra of the nucleoli and nucleus of HAEC cells in comparison with spectra of reference compounds of nucleobases.*

Figure S5. *The average Raman spectra of the perinuclear area of HAEC cells in comparison with reference standards.*

Figure S6. *The average Raman spectra of mitochondria of HAEC cells in comparison with selected standards.*

Figure S7. *Average Raman spectra of the cytoplasm of HAEC cells in comparison with spectra of selected standards.*

Figure S8. *Average Raman spectra of the cytoplasm and membrane of HAEC cells in comparison with spectra of selected standards.*

Figure S9. *Average Raman spectra of the cytoplasm and membrane of HAEC cells in comparison with spectra of selected standards in solid state.*

Table S1. *A list of reference compounds.*

Table S2. *Characteristic Raman bands of amino acids and proteins in the cytoplasm with detailed assignments.*

A list of abbreviations

A – adenine

ATP – adenosine triphosphate

C – cytosine

CEs – cholesterol esters

CHL AA – cholesteryl arachidonate

cyt. c – cytochrome c

cyt. b5 – cytochrome b5

cyt. P450 – cytochrome P450

DNA – deoxyribonucleic acid

ECs – endothelial cells

ER – endoplasmic reticulum

ETC – electron transport chain

FAs – fatty acids

G – guanine

HAEC cells – human aortic ECs cell line

KMC analysis – K-means cluster analysis

LDs – lipid droplets

OA – oleic acid

PA – palmitic acid

PC – phosphatidylcholine

PE – phosphatidylethanolamine
Phe – phenylalanine
pSer – O-phospho-L-serine
pThr – O-phospho-L-threonine
pTyr – O-phospho-L-tyrosine
RER – rough endoplasmic reticulum
RNA – ribonucleic acid
RS – Raman spectroscopy
Ser – L-serine
SER – smooth endoplasmic reticulum
SM – sphingomyelin
T – thymine
TAGs – triacylglycerols
Thr – L-threonine
TLN – trilinolein
TPA – tripalmitin
U – uracil

Supplementary Materials and Methods

Cells culture

For experiments, HAEC cells were grown in a microvascular EC growth medium (EGM-2MV, Lonza) supplemented with the necessary nutrients for their growth. Cells were seeded directly onto CaF_2 slides (Crystran Ltd) 24h before Raman imaging. Cell survival analysis using Trypan Blue was always performed at the time of seeding. The survival rate assessed in this manner was between 98% and 100%. This allowed the cells sufficient time to grow and spread, reaching an appropriate confluence (ca. 150,000 cells per slide). HAEC cells were grown and incubated at 37°C with continuously maintained humidity and a CO_2 content of 5%. Measurements were conducted on live cells in warm phosphate-buffered saline (PBS, Sigma Aldrich).

Data Analysis

Data processing of Raman data included removing artifacts from cosmic radiation, background subtraction, and reduction of residual autofluorescence (polynomial fitting, 3rd order). Subsequently, K-means cluster (KMC) analysis was employed to identify the major cellular structures, including the nucleus, nucleoli, perinuclear area (with contributions from the endoplasmic reticulum), mitochondria, lipid-rich area (LDs), cytoplasm, and cell membrane. KMC was carried out in the spectral ranges of 2650–3005 and 430–1775cm^{-1}. The goal was to identify seven classes; however, if this could not be achieved in a single step, KMC analysis was performed gradually. Initially, the cell region was separated from the background, and subsequent classes were then extracted based on the presence of a defined set of characteristic spectral bands. By utilizing both the fingerprint and high-wavenumber spectral regions, we were able to emphasize the presence of specific marker bands, rather than basing the classification of spectra solely on overall spectral intensity. After KMC, the average spectra for each cell class were extracted for further analysis. All spectra were presented in the spectral range of 500–3100 cm^{-1}. SI (**Figures S2-S9**) shows the original scale spectra with detailed assignments. An Origin 2023 software (OriginLab Corporation) was used for data visualization.

Raman measurements of references and data analysis

Raman spectra of amino acids in an aqueous solution were registered on a *Chiral*RAMAN-2X™ spectrometer (BioTools Inc.). The spectra were recorded in the 250–2500 cm^{-1} spectral range, with a 7 cm^{-1} resolution, an integration time of 1 s, and an excitation wavelength of 532 nm. The concentration and laser power were individually adjusted to each compound, i.e., L-serine (200 mM, 510 mW); O-phospho-L-serine (100 mM, 230 mW); L-threonine (100 mM, 260 mW); O-phospho-L-threonine (100 mM, 160 mW); O-phospho-L-tyrosine (25 mW, 180 mW). All solutions, before measurements, were passed through the Millex® (Merck Millipore™) syringe PTFE filters (pore size 0.45 μm) to eliminate solid impurities. Spectra were solvent- and baseline-corrected.

Single spectra were preprocessed using cosmic ray removal mode (filter size 3 and dynamic factor 8), then smoothed (Savitzky-Golay algorithm), baseline-corrected (Rubberband method with 64 points), and vector-normalized within the 500–3030 cm^{-1} range. Finally, the spectra of each compound were averaged. Pre-processing and analysis were performed using WITec Project FIVE 5.3 Plus software (WITec GmbH) and OPUS 7.0 software (Bruker Optik GmbH). Origin 2023 (OriginLab Corporation) was used for data presentation.

The list of reference compounds

Table S1 summarizes the neat compounds whose Raman spectra were used for the band assignment of the cell spectra. They were attributed to the cellular structures in which they are highly abundant.

Table S1*. A list of reference compounds.*

Cell compartment	Reference compound	Company
**Nucleus**	DNA from calf thymus	Sigma-Aldrich
	Ribonucleic acid from torula yeast	Sigma-Aldrich
	2-deoxy-D-ribose	Sigma-Aldrich
	D-(-)-ribose	Sigma-Aldrich
	Adenine	Sigma-Aldrich
	Cytosine	Sigma-Aldrich
	Guanine	Sigma-Aldrich
	Uracil	Sigma-Aldrich
	Thymine	Sigma-Aldrich
**Perinuclear area**	L-α-phosphatidylcholine	Sigma-Aldrich
	L-α-Phosphatidylethanolamine from egg yolk	Sigma-Aldrich
	Sphingomyelin	Sigma-Aldrich
**Mitochondria**	Cytochrome b5 human	Sigma-Aldrich
	Cytochrome p450 human	Sigma-Aldrich
	Cytochrome c, from bovine heart	Sigma-Aldrich
**Lipid droplets**	Palmitic acid	SigmaAldrich
	Oleic acid	Sigma-Aldrich
	Trilinolein	Supelco
	Tripalmitin	Supelco
	Cholesteryl arachidonate	Sigma Aldrich
**Cytoplasm**	albumin	Sigma-Aldrich
	L-Serine	Sigma-Aldrich
	O-phospho-L-serine	Sigma-Aldrich
	L-threonine	Sigma-Aldrich
	O-phospho-L-threonine	Sigma-Aldrich
	L-tyrosine	Sigma-Aldrich
	O-phospho-L-tyrosine	Sigma-Aldrich
	L-tryptophan	Sigma-Aldrich
	L-phenylalanine	Sigma-Aldrich

Fluorescence imaging

The presence of lipid droplets in HACEs was confirmed by HCS LipidTOX Deep Red Neutral Lipid Stain (LipidTOX, Invitrogen, Carlsbad, CA, USA) staining. Cell nuclei were counterstained with Hoechst 33342 (Invitrogen, Carlsbad, CA,F USA; 1:2000). LipidTOX was diluted at 1:200 in PBS solution and used on formalin-fixed cells. After 30 min of incubation in a mixture with Hoechst 33342 (1:2000), cells were washed twice and then stored in PBS solution during fluorescence measurements. Visualization of the mitochondria was performed by staining live HAECs with the MitoTracker™ Orange CM-H2TMRos (MitoTracker, Invitrogen, Carlsbad, CA, USA). HAECs were immersed in Live Cell Imaging Solution (Invitrogen, Carlsbad, CA, USA) and simultaneously added 500 nM of a MitoTracker and Hoechst 33342 (1:2000; Hoechst, Invitrogen, Carlsbad, CA, USA). Cells were incubated with dyes for 30 min at 37°C. Stained cells were washed twice with Live Cell Imaging Solution. Fluorescence measurements of HAECs were conducted using a Confocal Quantitative Image Cytometer (CQ, Yokogawa, Tokyo, Japan) equipped with a x20 magnification objective. The analysis was performed using ImageJ software (National Institutes of Health, Maryland, US).

The identification of individual cell organelles based on Raman imaging

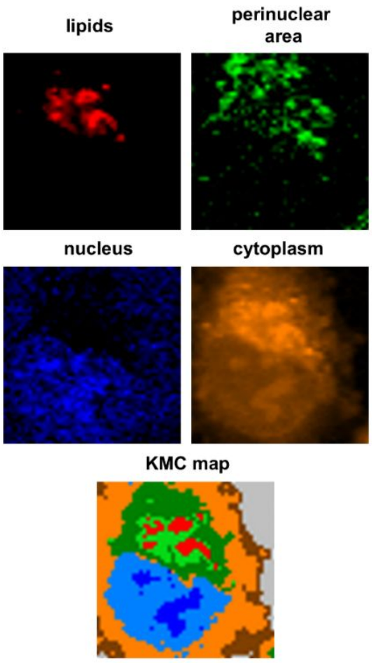

Figure S1. *KMC results of HAEC cell with representative Raman images integrated over 2820–2900 (lipids), 740–760 (perinuclear area containing ER and mitochondria), 780–800 (nucleus and nucleoli), and 2800–3050 cm* $^{-1}$ *(organic matter). Scale bar: 5 µm.*

The average spectra of subcellular components

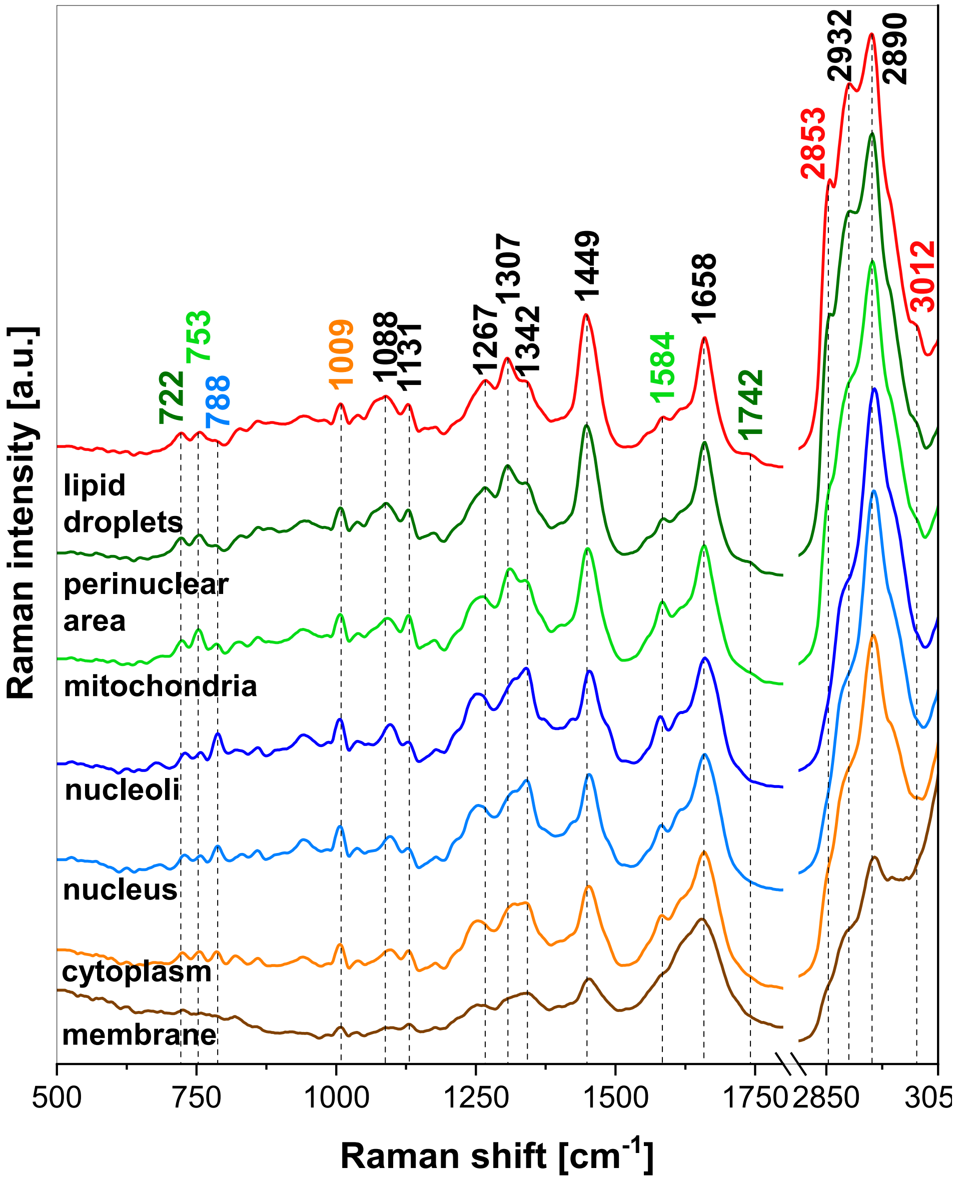

Figure S2. *The average spectra of subcellular components, including the nucleus (azure), nucleoli (blue), perinuclear area (green), mitochondria (vivid green), LDs (red), cytoplasm (orange), and cell membrane (dark brown), are magnified 3 times in the fingerprint region (500–1800 cm^{-1}) for clarity.*

Nucleus & nucleoli

The spatial organization of the nucleus observed in the Raman image is primarily attributed to its unique chemical composition. The nucleus comprises nucleic acids, including deoxyribonucleic acid (DNA) and ribonucleic acid (RNA), as well as an array of proteins, notably histones, which collectively form chromatin[1]. These constituents generate Raman signals that together produce the distinctive spectral profile of the nucleus (**Figure 2B**). Both DNA and RNA contain sugar moiety - deoxyribose in DNA or ribose in RNA – paired with a phosphate group that forms the sugar-phosphate backbone and one of five nitrogenous bases: adenine, cytosine, guanine, thymine, or uracil[2].

Spectroscopic analysis of DNA conformations

Benevides and Thomas (1983)[3] made a seminal contribution to understanding conformational differences in DNA using RS, demonstrating that distinct helical forms can be discerned by carefully analyzing specific vibrational markers[3–5]. Under physiological conditions, B-DNA is the predominant form, characterized by a C2′-endo sugar pucker and specific vibrational markers, such as symmetric phosphate stretching around 1090 cm^{-1} and a prominent band near 830 cm^{-1} (**Figures 2, S3** and **S4**). The A-DNA form exhibits a notable shift from 810 cm^{-1} to approximately 830 cm^{-1}, reflecting the adoption of a C3′-endo sugar pucker. When exposed to dehydration, high salt concentrations, certain alcohols, or osmotic stress (often mediated by changes in ionic strength and counterion type), B-DNA can transition to A-DNA. In A-DNA, the symmetric phosphate stretching band generally shifts to a higher frequency, typically around 1088 cm^{-1}; this reflects the altered backbone conformation associated with dehydration or the presence of specific solutes. Meanwhile, the band near 785 cm^{-1}, often present in both forms, may appear with reduced intensity or a slight shift. Bands associated with sugar-phosphate vibrations, such as those near 1480 cm^{-1}, can also become more pronounced in the A-form due to sugar puckering and backbone geometry differences. In contrast, under conditions of high salt, negative supercoiling, or the influence of specific binding proteins, DNA can adopt the left-handed Z-DNA conformation, reflected in notable changes in the Raman spectrum. These include a diminished or shifted band near 830 cm^{-1}, modified phosphate stretching modes, and the appearance of new markers such as bands near 625 cm^{-1} (G)[3,5]. A notable Raman band at 1422 cm^{-1}, generally attributed to the CH_2 scissoring or bending motions of the deoxyribose moiety, offers additional insight into the DNA conformational state; its intensity and precise position are sensitive to both conformation and hydration level, serving as a valuable complementary marker. In the B structure, a Raman band is detectable near 1422 cm^{-1}, previously associated with methylene deformations of the deoxyribose residue, and is sensitive to both the B-to-A and B-to-Z structural transitions[5,6]. Benevides et al. presented a detailed Raman spectroscopic investigation of Z-DNA, identifying key vibrational markers that distinguish this left-handed conformation from the more common B-DNA. The authors highlight a robust Z-backbone marker band around 745-750 cm^{-1}, which serves as the principal spectral indicator of the Z-DNA conformation in complex biological samples. Together, these subtle shifts in band positions and variations in intensity provide critical insights into the conformational state of nucleic acids within the nucleus. Such changes have been documented in studies exploring the biochemical effects of chemotherapeutic agents and other cellular stressors, thereby establishing RS as a powerful tool for monitoring drug-induced conformational dynamics in situ. For example, Haq Nawaz et al.[7] demonstrated that drug treatment induced measurable alterations in the Raman signatures of cancer cells, reflecting changes in nucleic acid conformation, hydration, and protein-nucleic acid interactions.

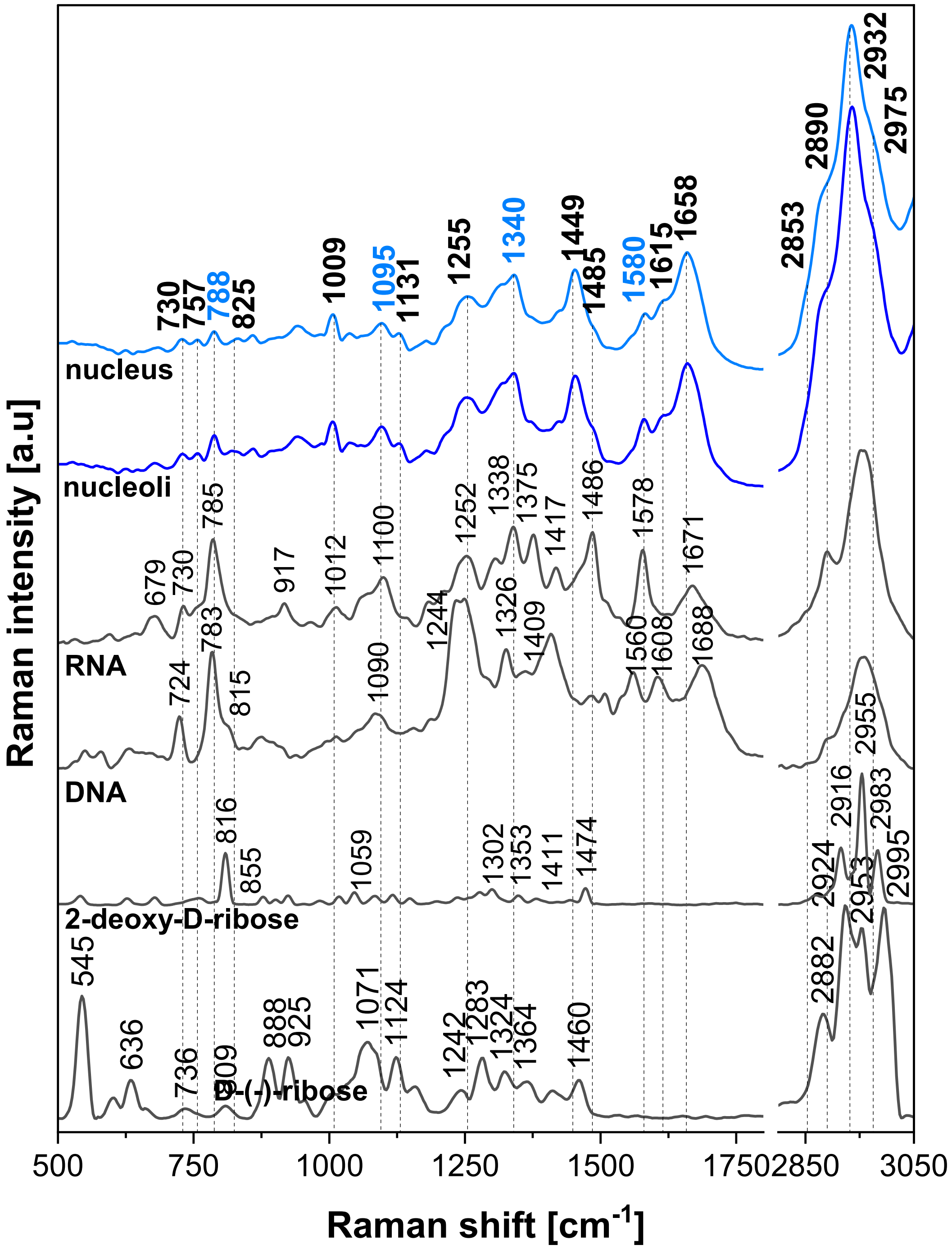

Figure S3. *Average Raman spectra of the nucleoli (blue) and nucleus of HAEC cells (azure) in comparison with spectra of reference compounds: calf thymus DNA, herring sperm DNA, Torula yeast, deoxy-2-D-ribose, and D-(-)-ribose.*

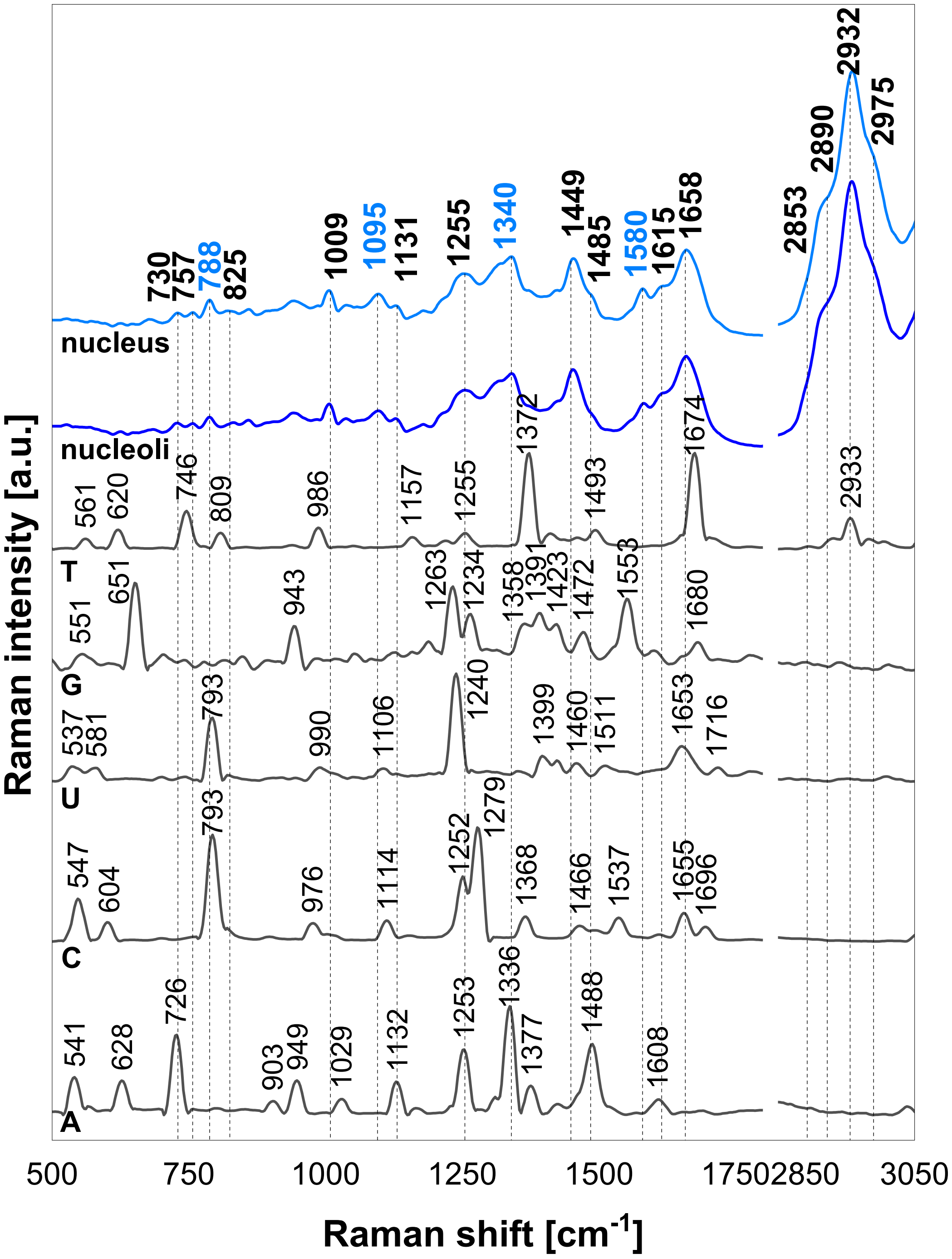

Figure S4. *Average Raman spectra of the nucleoli (blue) and nucleus of HAEC cells (azure) in comparison with spectra of reference compounds, five nucleobases: A, C, G, T, and U.*

Perinuclear area

The perinuclear area is lipid-rich and characterized by a significant involvement of the ER; other relevant components include the Golgi apparatus, lysosomes, lipids, and mitochondria. Here, the focus is on ER, a key cellular structure whose intricate lipid-protein composition poses a challenge for spectroscopy-based analysis[2]. Unlike LDs, which are almost entirely composed of lipids, or nuclei, which are rich in nucleic acids, the heterogeneous molecular composition of ER requires a more advanced approach for visualization. The notable spectral markers that facilitate the differentiation of

the ER from other perinuclear constituents are primarily related to increased phospholipid and sphingolipid content[8].

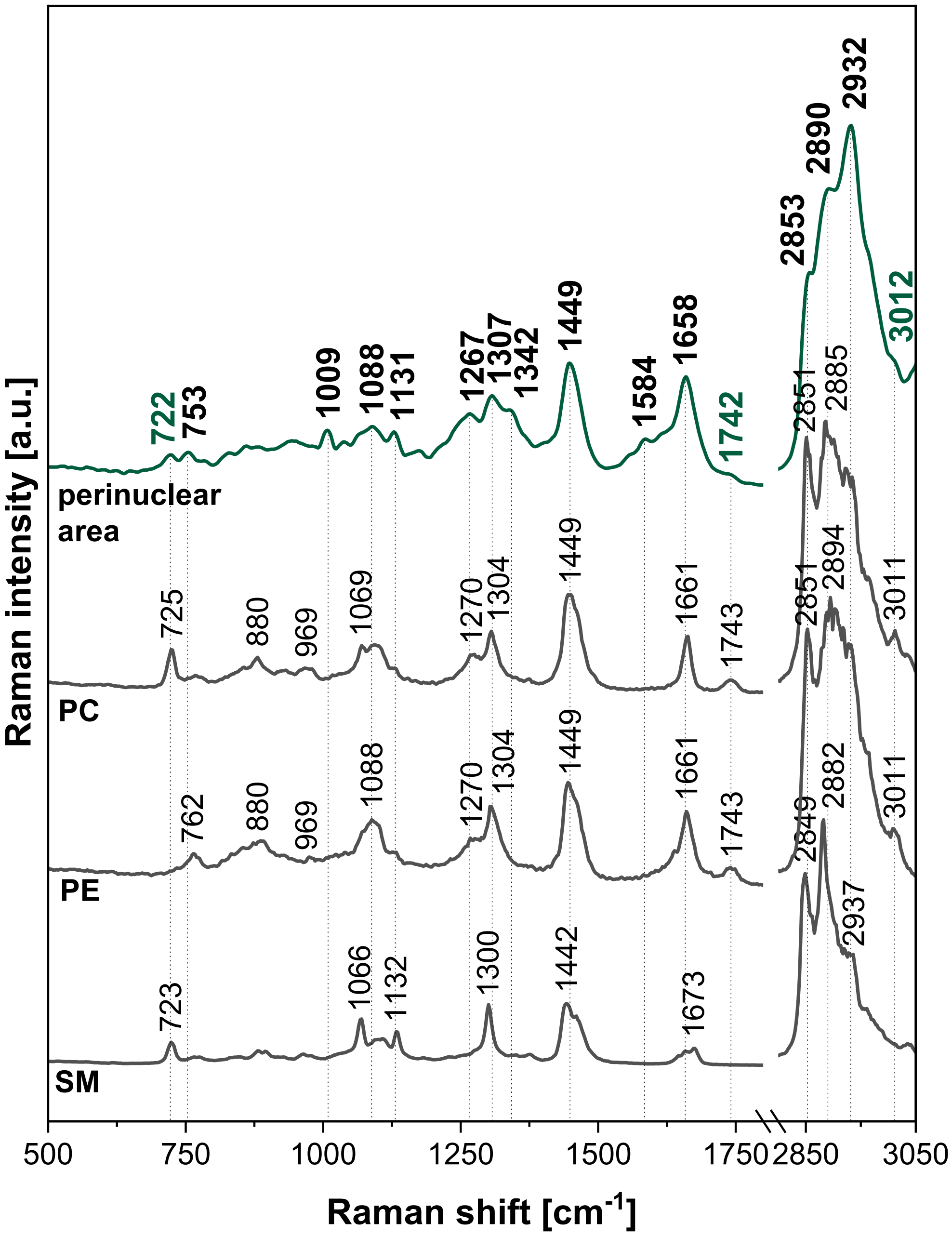

Figure S5. *The average Raman spectra of the perinuclear area (green) of HAEC cells in comparison with reference standards: phosphatidylcholine (PC), phosphatidylethanolamine (PE), and sphingomyelin (SM).*

Mitochondria

Mitochondria contain various proteins crucial for oxidative phosphorylation (Complexes I-V), with important roles played by heme-containing proteins, the cytochromes. Raman microscopy is sensitive enough to identify mitochondria based on a unique set of cytochrome signals related to vibrations of the pyrrole rings [9–11].

Resonance Raman Spectroscopy of hemoproteins

The RRS phenomenon occurs because significant Raman enhancement is observed when the laser excitation wavelength coincides with the absorption band of the porphyrin ring[10,12]. The Soret maxima of heme occur at 415-430 nm; therefore, excitation within this region provides strong enhancement, mainly of the ν4 band. However, the use of the excitation wavelength of 532 nm also allows for enhancement and tracking of the ν_{19} and ν_{10} bands, which fall within the Q band (530-550 nm) of the visible spectrum[13,14].

RRS provides insight into the oxidation and spin states[15,16] of the heme iron. For example, the ferric Fe^{3+} state in cyt. c undergoes a reduction process to the ferrous Fe^{2+} state during electron transport. From a spectroscopic perspective, this transition is efficiently tracked using a 405 nm excitation wavelength. A well-pronounced shift of the ν_4 porphyrin ring vibration mode is detected from around 1364 cm^{-1} (Fe^{2+} form) to around 1374 cm^{-1} (Fe^{3+} form)[17].

While RRS enhances cytochrome-specific bands, it can also raise the spectral background through fluorescence[18,19]. Excessive laser power may induce oxidation and other chemical modifications in cytochromes, evidenced by diminished or lost hemoprotein peaks at 753, 1131, 1307, and 1584 cm^{-1}, characteristic of reduced cytochromes[18,20–22]. The aldehyde-based fixation process may also induce the oxidation process of cytochromes, which are widely used to preserve cell structure and chemical composition[8,22].

Spectroscopic analysis of cytochromes

RS has also been used to track cytochrome interactions with other biological components, such as cardiolipin[23], and to identify iron heme ligands in different pH environments[24]. Studies of other heme-based proteins, such as hemoglobin[25], have further expanded our understanding of heme chemistry. Meanwhile, the intricate mitochondria-ER interplay mediated by cytochromes has broad implications. For instance, the translocation of cyt. c from mitochondria to the nucleus can influence tumor suppressor activity[26], while the localization of cyt. b_5 reductase (CBR1) between mitochondria and ER is vital for plant osmotic stress responses[27]. Ultimately, unraveling these complex interactions through cytochrome spectral signatures opens new avenues for research in cellular biology and could drive breakthroughs in fields ranging from cancer to agricultural science.

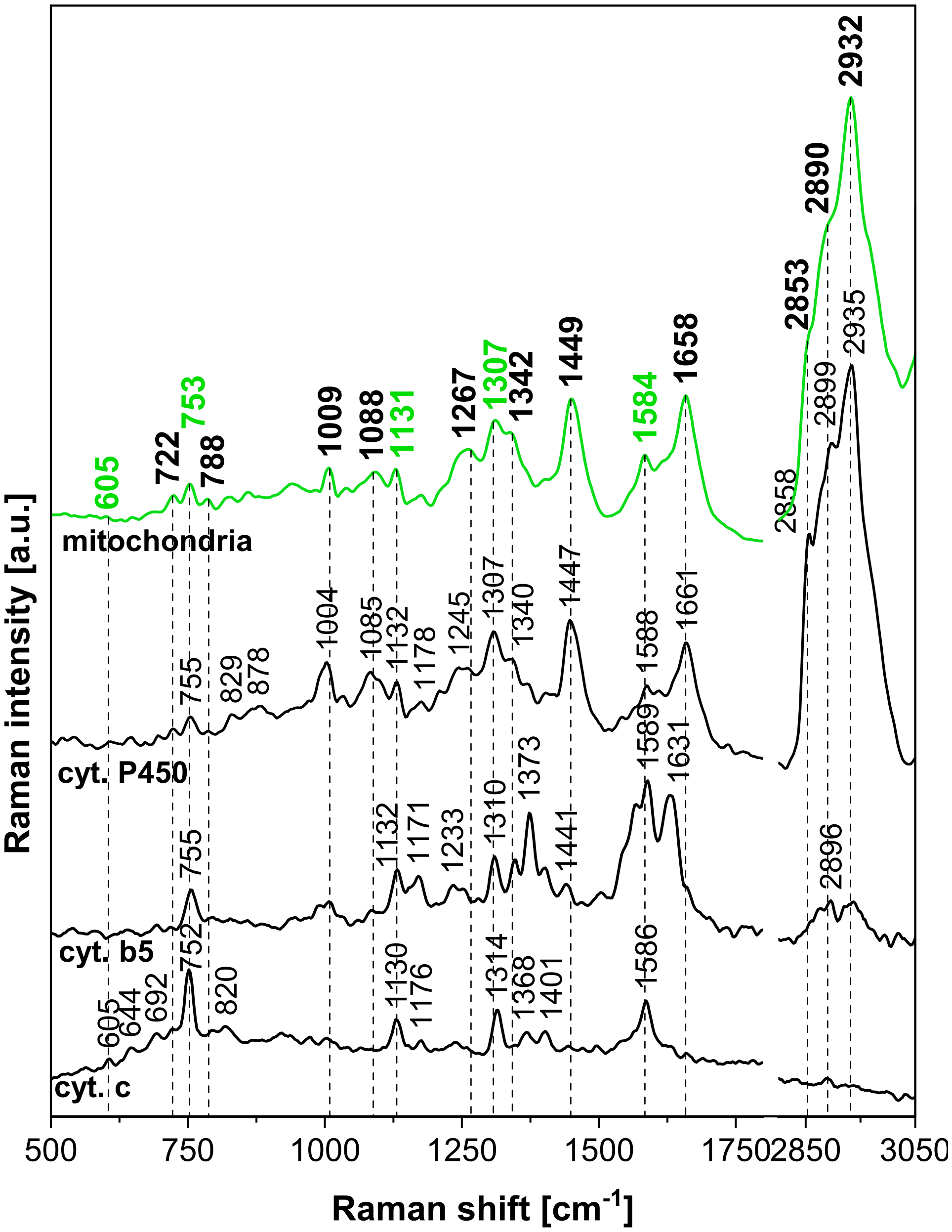

Figure S6. *The average Raman spectra of mitochondria (vivid green) of HAEC cells in comparison with selected standards: cytochrome P450 (cyt. P450), cytochrome b5 (cyt. b5), and cytochrome c (cyt. c).*

Lipid droplets

LDs are specialized organelles found in most eukaryotic cell types with various functions, including dynamic reservoirs for neutral lipids such as triglycerides and cholesteryl esters. RS has emerged as a label-free approach to investigate LD molecular composition and dynamics *in situ*, providing insights into cellular metabolism and lipid homeostasis. By analyzing CH-stretch and C=C signals, RS can detect changes in the unsaturation level of lipids, study LD-organelle interactions, and provide insight into metabolic adjustments under physiological or pathological conditions.

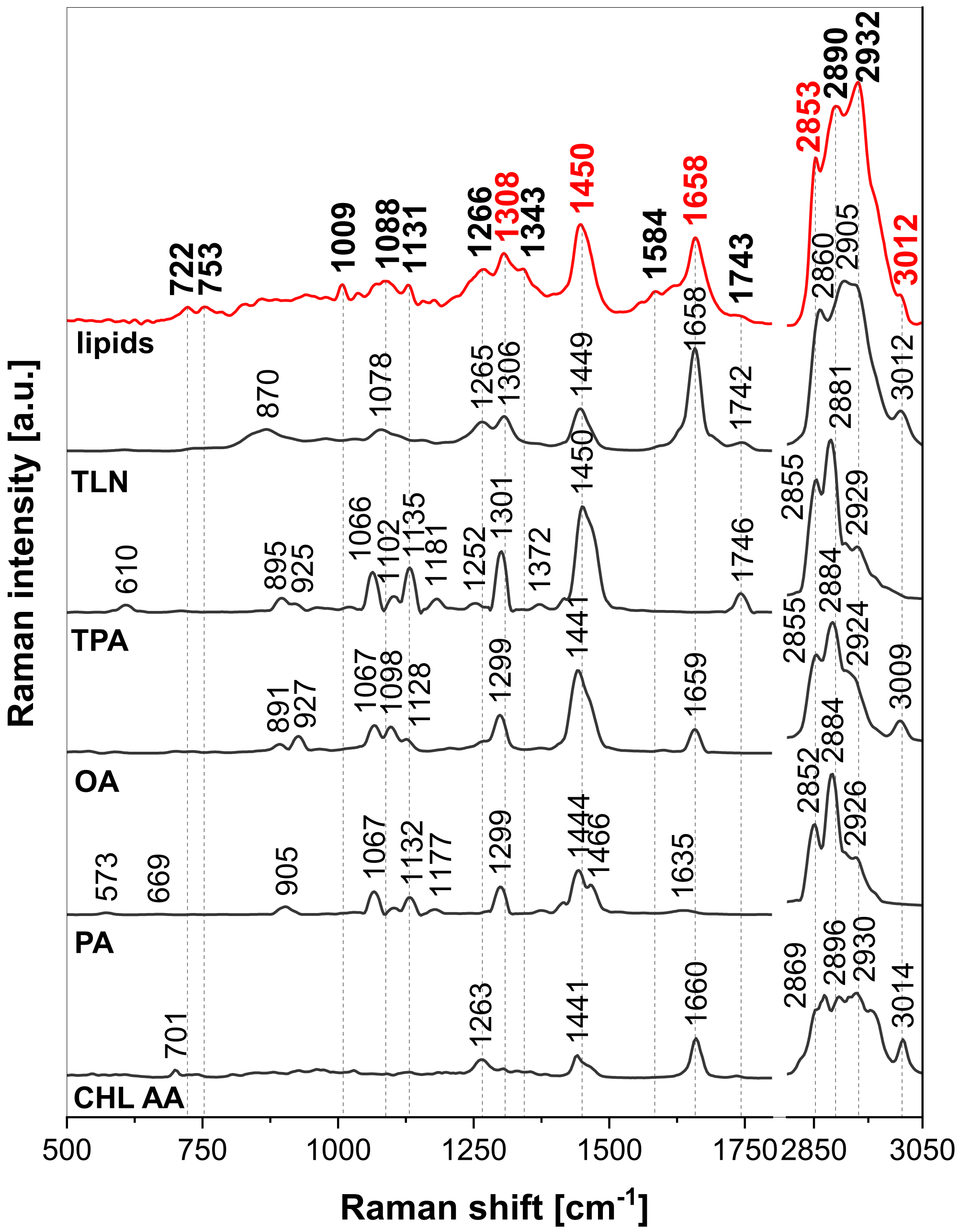

Figure S7. *The average Raman spectra of LDs (red) of HAEC cells in comparison with selected standards: trilinolein (TLN), tripalmitin (TPA), oleic acid (OA), palmitic acid (PA), and cholesteryl arachidonate (CHL AA).*

Cytoplasm

Occupying the intracellular space between the plasma membrane and the nucleus, the cytoplasm comprises a dense mixture of proteins, amino acids, FA, carbohydrates, mRNA, inorganic salts, and water[28–30]. Its aqueous component, the cytosol, has essential roles in metabolism, signal transduction, and structural organization. The cytoskeleton extends throughout the cytoplasm, anchoring organelles and enabling vesicular transport; its filaments are immersed in the cytosol. The cytosol serves as a medium for biochemical reactions, including glycolysis, gluconeogenesis, and partial steps of other metabolic reaction pathways[31]. Because protein signals often dominate the Raman spectra of the cytoplasm, we place particular emphasis on analyzing and assigning characteristic bands to albumin and

selected amino acids, which strongly contribute to the Raman profiles of cells. The importance of the cytoplasm in cellular function has spurred significant interest in using RS to study cytoplasmatic composition.

Spectroscopic analysis of the secondary structure of proteins

Raman studies of protein solutions and powders, which are used as model systems, provide insight into the structure of proteins[32,33]. The Raman spectra of proteins in solid states with different secondary structures have been previously described in detail by Rygula et al.[34]. Raman-based structural features can be determined by analyzing the vibrations of the amide bands, which can be resolved into nine normal modes designated as A, B, and I-VII, ordered by decreasing wavenumber. Among these, the amide I and III bands have been most extensively studied in isolated proteins and biological samples[34,35]. The positions and deconvolution of these bands suggest the presence of α helix structure at 1650-1665 (amide I) and 1265-1275 (amide III), as well as β-sheet structure at 1670-1680 (amide I) and 1230-1240 cm^{-1} (amide III).

Spectroscopic analysis of the structure of albumin

In the Raman spectrum of albumin, amide bands are visible at 1255 cm^{-1} (amide III) and 1658 (amide I), reflecting a secondary structure composition of roughly 55% α-helix and 45% β-sheet[36]. Among the amino acids mentioned in the article, in the structure of albumin, the most vivid is the presence of Phe (30 residues) based on bands at 1009, 1038, and 1610 cm^{-1}, together with Thr (30 residues) at 940 and 1410 cm^{-1}. Ser (22 residues) and Tyr (18 residues) can also be assigned based on Raman spectra at 940, 1342, and 1410 and 835, 850, and 1610 cm^{-1}, respectively.

Raman bands of amino acids

The Raman spectra of Ser and pSer show apparent differences (**Figure S8, Table S1**). The most prominent bands for Ser include unique signals at 815, 850 (ν_s(C–C–N) with a characteristic shoulder at 870 cm^{-1} [37]), and 1470 cm^{-1} ($\delta(CH_2)$[38]). In contrast, the Raman spectrum of pSer features an intense band at 1088 cm^{-1}, specific for PO_3^- vibrations[39]. The band intensity 815 increases, while 850 cm^{-1} is significantly reduced in pSer compared to Ser.

Thr shows characteristic Raman features at 560 cm^{-1} ($\rho(CO^{2-})$), 870 (ν_s(C–C–N)), , and 940[37] (**Figure S8**). Phosphorylation of Thr causes the band appearance specific to PO_3^- vibrations at 915 cm^{-1}, serving as a marker for pThr. Due to the low solubility of Tyr, its Raman spectrum was not obtained in solution. However, Raman spectra of amino acids in the solid state may exhibit differences in band intensities compared to the spectra of solutions. Nevertheless, in its solid state, Tyr (**Figure S9**) shows distinct features at 835, 1330, and 1615 cm^{-1}, which are related to ring vibrations (ν_{ring}) as the 645 cm^{-1} [40].

The influence of fixation on the molecular structure of proteins

Alcohol fixation leads to sample dehydration and protein precipitation, and significantly impacts lipids, which may dissolve. On the other hand, aldehyde fixation stabilizes structural and morphological details by forming intermolecular bridges between aldehyde groups and the reactive sites of biocomponents, such as amides and aromatic amino acids (such as Tyr and Phe). While preserving cellular architecture, this stabilization frequently induces conformational changes in proteins. Notably, an increase in the intensity of the amide I band has been observed during fixation by glutaraldehyde (GA) and formalin at several excitation wavelengths, including 488, 532, and 785 nm[8,41]. Additionally, GA fixation tends to increase the intensity of the 1040 cm^{-1} band while decreasing the intensity of the signal at 1005 cm^{-1}. This shift in the position of the Phe bands is a marker of conformational changes induced by interactions between GA and cellular proteins via aromatic amino acids[42,43]. Recent studies have shown that the cryopreservation of samples at 233 K enhances the signal-to-noise ratio, offering

better contrast for bands at 750 (cytochromes ν(pyr) breathing), 1680 (amide I), and 2850 cm^{-1} ($\nu(CH_2)$), and without damaging the sample due to crystallization[44].

Detection of lipid membranes using Raman spectroscopic methods

Other cytoplasm components, such as lipid membranes, restrict the cell's interior. However, the detection of membranes in a label-free manner is difficult due to their nanometer-scale dimension, structural diversity (a complex mix of proteins and heterogenous lipids), and dynamic modulation[45,46]. Several studies have focused on isolated models of lipid layers[47,48]. An alternative approach is the use of deuterium labeling or alkylation[49] to track extracellular lipids internalized by cells. For example, Yihiui et al. employed stimulated Raman scattering (SRS) to visualize the cellular distribution of palmitate labeled with deuterium in conjunction with a fluorescent palmitate analog (BODIPY-C12), to detect ER lipid membranes[50]. Similarly, the analysis of the accumulation of falcarinol, which contains an alkyne group, has been explored[51]. Yang et al.[52] applied a photo-zwitterionic polyene containing an ammonium cation and a sulfonate anion to target the plasma membrane through electrostatic interaction. Furthermore, techniques such as Surface-Enhanced Raman Spectroscopy (SERS) and Tip-Enhanced Raman Spectroscopy (TERS), which offer high specificity and spatial resolution, are invaluable tools for studying cellular membranes[53].

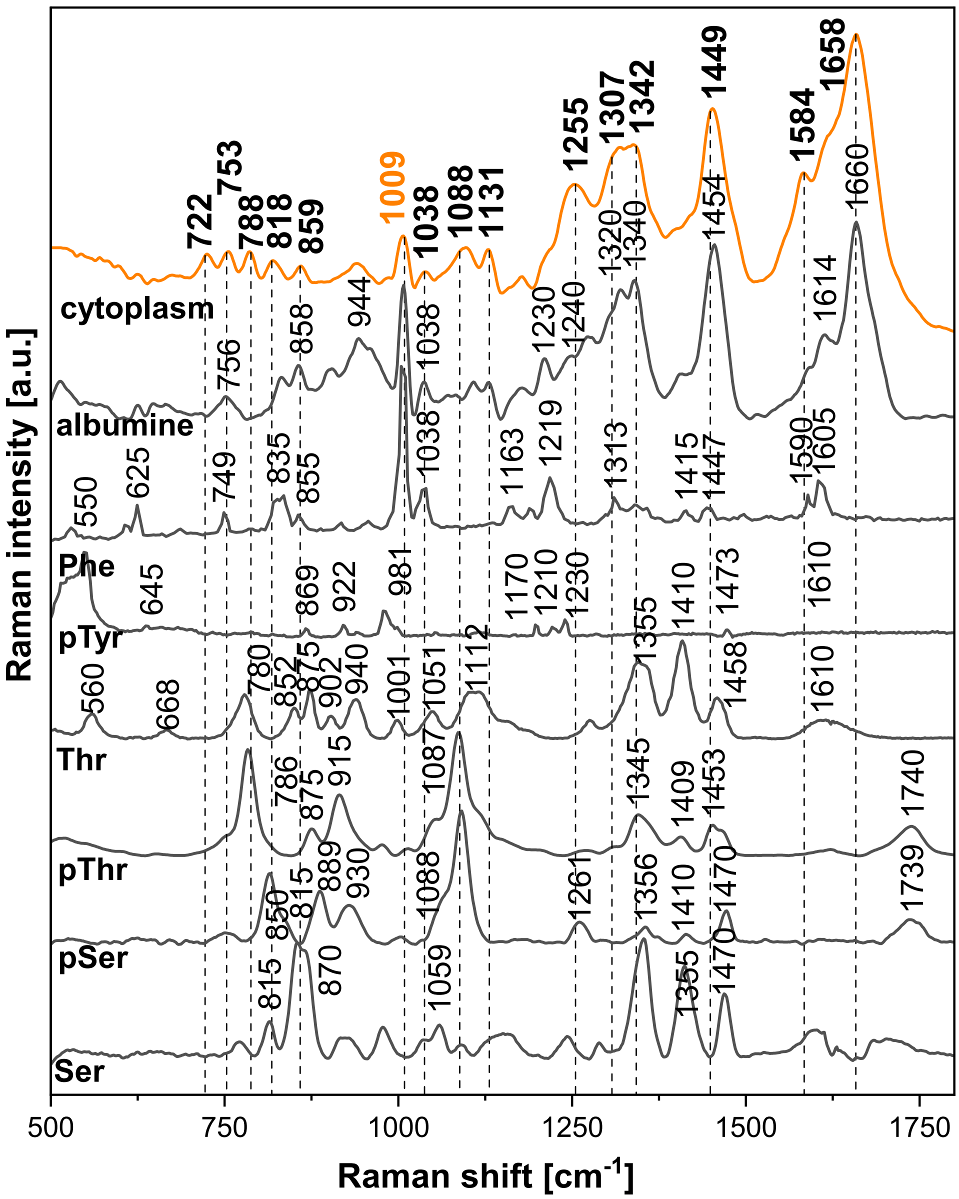

Figure S8. *Average Raman spectra of the cytoplasm (orange) of HAEC cells in comparison with spectra of O-phospho-L-tyrosine (pTyr), O-phospho-L-threonine (pThr), L-threonine (Thr), O-phospho-L-serine (pSer), and L-serine (Ser) in aqueous solution as well as with spectra of albumin and L-phenylalanine (Phe) in powder. The figure includes detailed assignments of bands specific to amino acids.*

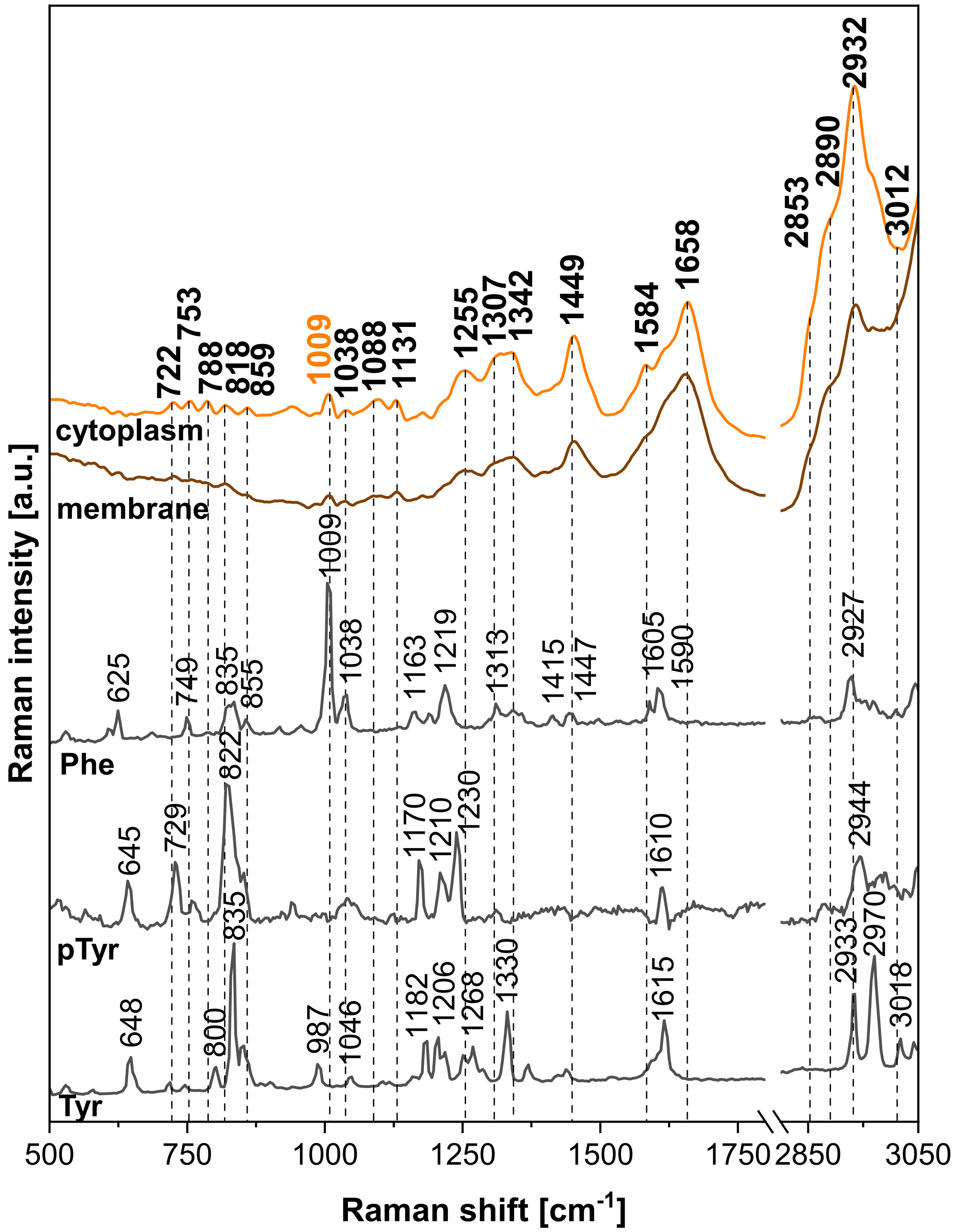

Figure S9. *The average Raman spectra of the cytoplasm (orange) and cell membrane (dark brown) of HAEC cells compared with spectra of the solid state of Phe, pTyr and Tyr. The figure includes detailed assignments of bands specific to amino acids.*

Table S2. *Characteristic Raman bands of amino acids and proteins in the cytoplasm with detailed assignments.*

Band [cm^{-1}]	Assignment	Reference
560	Thr ρ(CO^{2-}) (solution)	[37]
645	pTyr	
780	Thr (solution)	
785	pThr (solution)	
815	Ser, pSer ν(C–C–O) (solution)	[37]
835	Phe (solid-state), Tyr: Fermi doublet δ_{ring} (solution, solid state)	[54–56]

850	Ser ν_s(C–C–N) (solution), Tyr: Fermi doublet	37
870	Ser (solution)	
875	Thr ν_s(C–C–N) (solution)	
915	pThr (PO_3^-) (solution)	
930	pSer (solution)	
940	Thr (solution)	
**1009**	Phe ν_{ring} (solid state)	37
1038	Phe in-plane δ_{ring} (solid state)	37
1088	pSer PO_3^- (solution)	39,56
1170	pTyr	
1210	pTyr	
1230	amide III of albumin, pTyr	34
1240	amide III of albumin	34,35,37
1342	Thr δ(C–H)	37
1355	Ser, Thr	
1410	ν_s(COO^-) Ser, pSer, Thr	
1470	δ(CH_2) Ser, pSer	38
1610	ν_{ring} Tyr, pTyr	
1658	amide I of albumin	34,35,37

Abbreviations: β – bending, δ – deformation; ρ – rocking; ν – stretching (s – symmetric; as – asymmetric).

References

(1) Bonev, B.; Cavalli, G. Organization and Function of the 3D Genome. *Nat. Rev. Genet.* **2016**, *17* (11), 661–678. DOI: 10.1038/nrg.2016.112.

(2) Alberts, B.; Johnson, A.; Lewis, J.; Morgan, D.; Raff, M.; Roberts, K.; Walter, P. *Molecular Biology of the Cell*; Garland Science, 2017. DOI: 10.1201/9781315735368.

(3) Benevides, J. M.; Thomas, G. J. Characterization of DNA Structures by Raman Spectroscopy: High-Salt and Low-Salt Forms of Double Helical Poly(DG-DC) in H2O and D2O Solutions and Application to B, Z and A-DNA. *Nucleic Acids Res.* **1983**, *11* (16), 5747–5761. DOI: 10.1093/nar/11.16.5747.

(4) Thomas, G. J.; Benevides, J. M.; Overman, S. A.; Ueda, T.; Ushizawa, K.; Saitoh, M.; Tsuboi, M. Polarized Raman Spectra of Oriented Fibers of A DNA and B DNA: Anisotropic and Isotropic Local Raman Tensors of Base and Backbone Vibrations. *Biophys. J.* **1995**, *68* (3), 1073–1088. DOI: 10.1016/S0006-3495(95)80282-1.

(5) Warted, R. M.; Harrell, J. T.; Zacharias, W.; Wells, R. D. Raman Spectroscopy Study of the B-z Transition in (Dg-Dc)n o (Dg-Dc)n and a Dna Restriction Fragment. *J. Biomol. Struct. Dyn.* **1983**, *1* (1), 83–96. DOI: 10.1080/07391102.1983.10507427.

(6) Prescott, B.; Steinmetz, W.; Thomas, G. J.; Thomas Jr, G. J. Characterization of DNA Structures by Laser Raman Spectroscopy. *Biopolymers* **1984**, *23* (2), 235–256. DOI: 10.1002/bip.360230206.

(7) Nawaz, H.; Garcia, A.; Meade, A. D.; Lyng, F. M.; Byrne, H. J. Raman Micro Spectroscopy Study of the Interaction of Vincristine with A549 Cells Supported by Expression Analysis of Bcl-2 Protein. *Analyst* **2013**, *138* (20), 6177–6184. DOI: 10.1039/c3an00975k.

(8) Bik, E.; Dorosz, A.; Mateuszuk, L.; Baranska, M.; Majzner, K. Fixed versus Live Endothelial Cells: The Effect of Glutaraldehyde Fixation Manifested by Characteristic Bands on the Raman Spectra of Cells. *Spectrochim. Acta - Part A Mol. Biomol. Spectrosc.* **2020**, *240*, 118460. DOI: 10.1016/j.saa.2020.118460.

(9) Morimoto, T.; Chiu, L.; Kanda, H.; Kawagoe, H.; Ozawa, T.; Nakamura, M.; Nishida, K.; Fujita, K.; Fujikado, T. Using Redox-Sensitive Mitochondrial Cytochrome Raman Bands for Label-Free Detection of Mitochondrial Dysfunction. *Analyst* **2019**, *144* (8), 2531–2540. DOI: 10.1039/C8AN02213E.

(10) Bik, E.; Mateuszuk, L.; Stojak, M.; Chlopicki, S.; Baranska, M.; Majzner, K. Menadione-Induced Endothelial Inflammation Detected by Raman Spectroscopy. *Biochim. Biophys. Acta - Mol. Cell Res.* **2021**, *1868* (2), 118911. DOI: 10.1016/j.bbamcr.2020.118911.

(11) Ptaszek, M. Rational Design of Fluorophores for in Vivo Applications. In *Progress in Molecular Biology and Translational Science*; Morris, M. C. B. T.-P. in M. B. and T. S., Ed.; Academic Press, 2013; Vol. 113, pp 59–108. DOI: 10.1016/B978-0-12-386932-6.00003-X.

(12) Zhu, J.; Zhu, J.; Xie, H.; Tang, J.; Miao, Y.; Cai, L.; Hildebrandt, P.; Han, X. X. In Situ Raman Spectroscopy Reveals Cytochrome c Redox-Controlled Modulation of Mitochondrial Membrane Permeabilization That Triggers Apoptosis. *Nano Lett.* **2024**, *24* (1), 370–377. DOI: 10.1021/acs.nanolett.3c04129.

(13) Chen, Z.; Liu, J.; Tian, L.; Zhang, Q.; Guan, Y.; Chen, L.; Liu, G.; Yu, H.; Tian, Y.; Huang, Q. Raman Micro-Spectroscopy Monitoring of Cytochrome c Redox State in Candida Utilis during Cell Death under Low-Temperature Plasma-Induced Oxidative Stress. *Analyst* **2020**, *145* (11), 3922–3930. DOI: 10.1039/D0AN00507J.

(14) Mukherjee, R.; Verma, T.; Nandi, D.; Umapathy, S. Identification of a Resonance Raman Marker for Cytochrome to Monitor Stress Responses in Escherichia Coli. *Anal. Bioanal. Chem.* **2020**, *412* (22), 5379–5388. DOI: 10.1007/s00216-020-02753-y.

(15) Spiro, T. G.; Strekas, T. C. Resonance Raman Spectra of Heme Proteins. Effects of Oxidation and Spin State. *J. Am. Chem. Soc.* **1974**, *96* (2), 338–345. DOI: 10.1021/ja00809a004.

(16) Dorosz, A.; Grosicki, M.; Dybas, J.; Matuszyk, E.; Rodewald, M.; Meyer, T.; Popp, J.; Malek, K.; Baranska, M. Eosinophils and Neutrophils-Molecular Differences Revealed by Spontaneous Raman, CARS and Fluorescence Microscopy. *Cells* **2020**, *9* (9). DOI: 10.3390/cells9092041.

(17) Szczesny-Malysiak, E.; Bartkowiak, A.; Dybas, J. Label-Free Tracking of Cytochrome C Oxidation State in Live Cells by Resonance Raman Imaging. *FEBS Lett.* **2024**, *598* (16), 1981–1988. DOI: 10.1002/1873-3468.14905.

(18) Virdis, B.; Millo, D.; Donose, B. C.; Batstone, D. J. Real-Time Measurements of the Redox States of c-Type Cytochromes in Electroactive Biofilms: A Confocal Resonance Raman Microscopy Study. *PLoS One* **2014**, *9* (2), e89918. DOI: 10.1371/journal.pone.0089918.

(19) Hu, S.; Morris, I. K.; Singh, J. P.; Smith, K. M.; Spiro, T. G. Complete Assignment of Cytochrome c Resonance Raman Spectra via Enzymic Reconstitution with Isotopically Labeled Hemes. *J. Am. Chem. Soc.* **1993**, *115* (26), 12446–12458. DOI: 10.1021/ja00079a028.

(20) Kakita, M.; Okuno, M.; Hamaguchi, H. Quantitative Analysis of the Redox States of Cytochromes in a Living L929 (NCTC) Cell by Resonance Raman Microspectroscopy. *J. Biophotonics* **2013**, *6* (3), 256–259. DOI: 10.1002/jbio.201200042.

(21) Hildebrandt, P.; Heimburg, T.; Marsh, D.; Powell, G. L. Conformational Changes in Cytochrome c and Cytochrome Oxidase upon Complex Formation: A Resonance Raman Study. *Biochemistry* **1990**, *29* (6), 1661–1668. DOI: 10.1021/bi00458a044.

(22) Okada, M.; Smith, N. I.; Palonpon, A. F.; Endo, H.; Kawata, S.; Sodeoka, M.; Fujita, K. Label-Free Raman Observation of Cytochrome c Dynamics during Apoptosis. *Proc. Natl. Acad. Sci. U. S. A.* **2012**, *109* (1), 28–32. DOI: 10.1073/pnas.1107524108.

(23) Zhu, J.; Jiang, M.; Ma, H.; Zhang, H.; Cheng, W.; Li, J.; Cai, L.; Han, X. X.; Zhao, B. Redox-State-Mediated Regulation of Cytochrome c Release in Apoptosis Revealed by Surface-

Enhanced Raman Scattering on Nickel Substrates. *Angew. Chemie Int. Ed.* **2019**, *58* (46), 16499–16503. DOI: 10.1002/anie.201909638.

(24) Milazzo, L.; Tognaccini, L.; Howes, B. D.; Smulevich, G. Probing the Non-Native States of Cytochrome c with Resonance Raman Spectroscopy: A Tool for Investigating the Structure–Function Relationship. *J. Raman Spectrosc.* **2018**, *49* (6), 1041–1055. DOI: 10.1002/jrs.5315.

(25) Dybas, J.; Bokamper, M. J.; Marzec, K. M.; Mak, P. J. Probing the Structure-Function Relationship of Hemoglobin in Living Human Red Blood Cells. *Spectrochim. Acta. A. Mol. Biomol. Spectrosc.* **2020**, *239*, 118530. DOI: 10.1016/j.saa.2020.118530.

(26) González-Arzola, K.; Díaz-Quintana, A.; Bernardo-García, N.; Martínez-Fábregas, J.; Rivero-Rodríguez, F.; Casado-Combreras, M. Á.; Elena-Real, C. A.; Velázquez-Cruz, A.; Gil-Caballero, S.; Velázquez-Campoy, A.; Szulc, E.; Gavilán, M. P.; Ayala, I.; Arranz, R.; Ríos, R. M.; Salvatella, X.; Valpuesta, J. M.; Hermoso, J. A.; De la Rosa, M. A.; Díaz-Moreno, I. Nucleus-Translocated Mitochondrial Cytochrome c Liberates Nucleophosmin-Sequestered ARF Tumor Suppressor by Changing Nucleolar Liquid–Liquid Phase Separation. *Nat. Struct. Mol. Biol.* **2022**, *29* (10), 1024–1036. DOI: 10.1038/s41594-022-00842-3.

(27) Xiao, R.; Youngjun, O.; Zhang, X.; Thi, N. T. O. N.; Lu, H.; Hwang, I. Osmotic Stress-Induced Localisation Switch of CBR1 from Mitochondria to the Endoplasmic Reticulum Triggers ATP Production via β-Oxidation to Respond to Osmotic Shock. *Plant Cell Environ.* **2023**, *46* (11), 3420–3432. DOI: 10.1111/pce.14671.

(28) Carlson, B. M. Cells. In *The Human Body*; Carlson, B. M. B. T.-T. H. B., Ed.; Academic Press, 2019; pp 1–25. DOI: 10.1016/b978-0-12-804254-0.00001-6.

(29) Pagliaro, L. Mechanisms for Cytoplasmic Organization: An Overview. In *Microcompartmentation and Phase Separation in Cytoplasm*; Walter, H., Brooks, D. E., Srere, P. A. B. T.-I. R. of C., Eds.; Academic Press, 1999; Vol. 192, pp 303–318. DOI: 10.1016/S0074-7696(08)60531-8.

(30) Hunter, T. The Genesis of Tyrosine Phosphorylation. *Cold Spring Harb. Perspect. Biol.* **2014**, *6* (5), a020644. DOI: 10.1101/cshperspect.a020644.

(31) Cooper, G. M. Pathways of Intracellular Signal Transduction. In *The Cell: A Molecular Approach*; 2013; pp 608–614.

(32) Hauptmann, A.; Hoelzl, G.; Mueller, M.; Bechtold-Peters, K.; Loerting, T. Raman Marker Bands for Secondary Structure Changes of Frozen Therapeutic Monoclonal Antibody Formulations During Thawing. *J. Pharm. Sci.* **2023**, *112* (1), 51–60. DOI: 10.1016/j.xphs.2022.10.015.

(33) Wilkosz, N.; Czaja, M.; Seweryn, S.; Skirlińska-Nosek, K.; Szymonski, M.; Lipiec, E.; Sofińska, K. Molecular Spectroscopic Markers of Abnormal Protein Aggregation. *Molecules* **2020**, *25* (11). DOI: 10.3390/molecules25112498.

(34) Rygula, A.; Majzner, K.; Marzec, K. M.; Kaczor, A.; Pilarczyk, M.; Baranska, M. Raman Spectroscopy of Proteins: A Review. *J. Raman Spectrosc.* **2013**, *44* (8), 1061–1076. DOI: 10.1002/jrs.4335.

(35) Nemecek, D.; Stepanek, J.; Thomas, G. J. Raman Spectroscopy of Proteins and Nucleoproteins. *Curr. Protoc. Protein Sci.* **2013**, *71* (SUPPL.71), 17.8.1-17.8.52. DOI: 10.1002/0471140864.ps1708s71.

(36) Majumder, A. B.; Gupta, S.; Majumder, S.; Singh, D. A Heart Disease Prediction Model Using Merged XGBoost-SVM Classifier and Particle Swarm Optimization. In *2024 5th International Conference on Mobile Computing and Sustainable Informatics (ICMCSI)*; 2024; pp 241–248. DOI: 10.1109/ICMCSI61536.2024.00042.

(37) Zhu, G.; Zhu, X.; Fan, Q.; Wan, X. Raman Spectra of Amino Acids and Their Aqueous Solutions.

Spectrochim. Acta Part A Mol. Biomol. Spectrosc. **2011**, *78* (3), 1187–1195. DOI: 10.1016/j.saa.2010.12.079.

(38) Jarmelo, S.; Carey, P. R.; Fausto, R. The Raman Spectra of Serine and 3,3-Dideutero-Serine in Aqueous Solution. *Vib. Spectrosc.* **2007**, *43* (1), 104–110. DOI: 10.1016/j.vibspec.2006.06.021.

(39) Xie, Y.; Jiang, Y.; Ben-Amotz, D. Detection of Amino Acid and Peptide Phosphate Protonation Using Raman Spectroscopy. *Anal. Biochem.* **2005**, *343* (2), 223–230. DOI: 10.1016/j.ab.2005.05.038.

(40) Hernández, B.; Coïc, Y. M.; Pflüger, F.; Kruglik, S. G.; Ghomi, M. All Characteristic Raman Markers of Tyrosine and Tyrosinate Originate from Phenol Ring Fundamental Vibrations. *J. Raman Spectrosc.* **2016**, *47* (2), 210–220. DOI: 10.1002/jrs.4776.

(41) Rangan, S.; Wong, R.; Schulze, H. G.; Vardaki, M. Z.; Blades, M. W.; Turner, R. F. B.; Piret, J. M. Saline Dry Fixation for Improved Cell Composition Analysis Using Raman Spectroscopy. *Analyst* **2023**, *148* (12), 2745–2757. DOI: 10.1039/D2AN01916G.

(42) Bulat, K.; Dybas, J.; Kaczmarska, M.; Rygula, A.; Jasztal, A.; Szczesny-Malysiak, E.; Baranska, M.; Wood, B. R.; Marzec, K. M. Multimodal Detection and Analysis of a New Type of Advanced Heinz Body-like Aggregate (AHBA) and Cytoskeleton Deformation in Human RBCs. *Analyst* **2020**, *145* (5), 1749–1758. DOI: 10.1039/c9an01707k.

(43) Hernández, B.; Pflüger, F.; Kruglik, S. G.; Ghomi, M. Characteristic Raman Lines of Phenylalanine Analyzed by a Multiconformational Approach. *J. Raman Spectrosc.* **2013**, *44* (6), 827–833. DOI: 10.1002/jrs.4290.

(44) Mizushima, K.; Kumamoto, Y.; Tamura, S.; Yamanaka, M.; Mochizuki, K.; Li, M.; Egoshi, S.; Dodo, K.; Harada, Y.; Smith, N. I.; Sodeoka, M.; Tanaka, H.; Fujita, K. Raman Microscopy of Cryofixed Biological Specimens for High-Resolution and High-Sensitivity Chemical Imaging. *Sci. Adv.* **2025**, *10* (50), eadn0110. DOI: 10.1126/sciadv.adn0110.

(45) Shen, Y.; Wei, L.; Min, W. Raman Imaging Reveals Insights into Membrane Phase Biophysics in Cells. *J. Phys. Chem. B* **2023**, *127* (28), 6233–6240. DOI: 10.1021/acs.jpcb.3c03125.

(46) Uematsu, M.; Shimizu, T. Raman Microscopy-Based Quantification of the Physical Properties of Intracellular Lipids. *Commun. Biol.* **2021**, *4* (1), 1176. DOI: 10.1038/s42003-021-02679-w.

(47) Collard, L.; Sinjab, F.; Notingher, I. Raman Spectroscopy Study of Curvature-Mediated Lipid Packing and Sorting in Single Lipid Vesicles. *Biophys. J.* **2019**, *117* (9), 1589–1598. DOI: 10.1016/j.bpj.2019.09.020.

(48) Nahmad-Rohen, A.; Regan, D.; Masia, F.; McPhee, C.; Pope, I.; Langbein, W.; Borri, P. Quantitative Label-Free Imaging of Lipid Domains in Single Bilayers by Hyperspectral Coherent Raman Scattering. *Anal. Chem.* **2020**, *92* (21), 14657–14666. DOI: 10.1021/acs.analchem.0c03179.

(49) Song, D. Y.; Stubbe, J.; Nocera, D. G. Protein Engineering a PhotoRNR Chimera Based on a Unifying Evolutionary Apparatus among the Natural Classes of Ribonucleotide Reductases. *Proc. Natl. Acad. Sci.* **2024**, *121* (18), e2317291121. DOI: 10.1073/pnas.2317291121.

(50) Fujita, K.; Mizushima, K.; Tamura, S.; Yamanaka, M.; Li, M.; Harada, Y.; Smith, N. I.; Kumamoto, Y.; Tanaka, H. High-Sensitivity Raman Microscopy of Cryofixed Biological Samples. In *Proc.SPIE*; 2024; Vol. PC12839, p PC1283908. DOI: 10.1117/12.3000955.

(51) Matuszyk, E.; Adamczyk, A.; Radwan, B.; Pieczara, A.; Szcześniak, P.; Mlynarski, J.; Kamińska, K.; Baranska, M. Multiplex Raman Imaging of Organelles in Endothelial Cells. *Spectrochim. Acta - Part A Mol. Biomol. Spectrosc.* **2021**, *255*, 119658. DOI: 10.1016/j.saa.2021.119658.

(52) Yang, Y.; Bai, X.; Hu, F. Photoswitchable Polyynes for Multiplexed Stimulated Raman Scattering Microscopy with Reversible Light Control. *Nat. Commun.* **2024**, *15* (1), 2578. DOI: 10.1038/s41467-024-46904-6.

(53) Mrđenović, D.; Tang, Z.-X.; Pandey, Y.; Su, W.; Zhang, Y.; Kumar, N.; Zenobi, R. Regioselective Tip-Enhanced Raman Spectroscopy of Lipid Membranes with Sub-Nanometer Axial Resolution. *Nano Lett.* **2023**, *23* (9), 3939–3946. DOI: 10.1021/acs.nanolett.3c00689.

(54) De Gelder, J.; De Gussem, K.; Vandenabeele, P.; Moens, L. Reference Database of Raman Spectra of Biological Molecules. *J. Raman Spectrosc.* **2007**, *38* (9), 1133–1147. DOI: 10.1002/jrs.1734.

(55) Jenkins, A. L.; Larsen, R. A.; Williams, T. B. Characterization of Amino Acids Using Raman Spectroscopy. *Spectrochim. Acta. A. Mol. Biomol. Spectrosc.* **2005**, *61* (7), 1585–1594. DOI: 10.1016/j.saa.2004.11.055.

(56) Abramczyk, H.; Imiela, A.; Brożek-Płuska, B.; Kopeć, M.; Surmacki, J.; Śliwińska, A. Aberrant Protein Phosphorylation in Cancer by Using Raman Biomarkers. *Cancers.* **2019**, *11* (12). DOI: 10.3390/cancers11122017.
